# Heterosis analysis and underlying molecular regulatory mechanism in a wide-compatible neo-tetraploid rice line with long panicles

**DOI:** 10.1186/s12870-020-2291-z

**Published:** 2020-02-21

**Authors:** Mohammed Abdullah Abdulraheem Ghaleb, Cong Li, Muhammad Qasim Shahid, Hang Yu, Junhong Liang, Ruoxin Chen, Jinwen Wu, Xiangdong Liu

**Affiliations:** 1 State Key Laboratory for Conservation and Utilization of Subtropical Agro-Bioresources, South China Agricultural University, Guangzhou, 510642 China; 2 Guangdong Provincial Key Laboratory of Plant Molecular Breeding, South China Agricultural University, Guangzhou, 510642 China; 3 College of Agriculture, South China Agricultural University, Guangzhou, 510642 China; 4 Guangdong Laboratory for Lingnan Modern Agriculture, Guangzhou, 510642 China

**Keywords:** Rice (*Oryza sativa* L.), Neo-tetraploid rice, Hybrid vigor, Panicle, Transcriptome

## Abstract

**Background:**

Neo-tetraploid rice, which is a new germplasm developed from autotetraploid rice, has a powerful biological and yield potential and could be used for commercial utilization. The length of panicle, as a part of rice panicle architecture, contributes greatly to high yield. However, little information about long panicle associated with heterosis or hybrid vigor is available in neo-tetraploid rice.

**Results:**

In the present study, we developed a neo-tetraploid rice line, Huaduo 8 (H8), with long panicles and harboring wide-compatibility genes for pollen and embryo sac fertility. All the hybrids generated by H8 produced significant high-parent yield heterosis and displayed long panicles similar to H8. RNA-seq analysis detected a total of 4013, 7050, 6787 and 6195 differentially expressed genes uniquely belonging to F_1_ and specifically (DEGFu-sp) associated with leaf, sheath, main panicle axis and spikelet in the two hybrids, respectively. Of these DEGFu-sp, 279 and 89 genes were involved in kinase and synthase, and 714 cloned genes, such as *GW8*, *OsGA20ox1*, *Ghd8*, *GW6a*, and *LP1*, were identified and validated by qRT-PCR. A total of 2925 known QTLs intervals, with an average of 1~100 genes per interval, were detected in both hybrids. Of these, 109 yield-related QTLs were associated with seven important traits in rice. Moreover, 1393 non-additive DEGs, including 766 up-regulated and 627 down-regulated, were detected in both hybrids. Importantly, eight up-regulated genes associated with panicle were detected in young panicles of the two hybrids compared to their parents by qRT-PCR. Re-sequencing analysis depicted that *LP* (a gene controlling long panicle) sequence of H8 was different from many other neo-tetraploid rice and most of the diploid and autotetraploid lines. The qRT-PCR results showed that *LP* was up-regulated in the hybrid compared to its parents at very young stage of panicle development.

**Conclusions:**

These results suggested that H8 could overcome the intersubspecific autotetraploid hybrid rice sterility caused by embryo sac and pollen sterility loci. Notably, long panicles of H8 showed dominance phenomenon and played an important role in yield heterosis, which is a complex molecular mechanism. The neo-tetraploid rice is a useful germplasm to attain high yield of polyploid rice.

## Background

Rice is one of the world’s most important cereal grains [1]. Hybrid rice occupies more than 50% of the total rice area in China, and has a yield advantage of about 10–20% over inbred varieties [2]. However, with an increasing world population and gradually deteriorating environment, food security has become a major challenge, especially in Asia and Africa [3, 4]. During the last few years, rice production is stagnant due to intensification of crops and other biotic and abiotic factors. Therefore, it is of immense importance to develop high yielding rice cultivars resistant to various biotic and abiotic stresses.

Autotetraploid rice is a useful germplasm developed from diploid rice by chromosome doubling using colchicine treatment. Intersubspecific hybrids of autotetraploid rice have a powerful biological and yield potential, which may become a new way to breed rice in future [5–7]. However, low seed setting is the major hindrance in the use of autotetraploid rice at commercial level [8–13]. Polyploidy fortifies F_1_ pollen sterility loci interactions, which causes meiosis abnormalities and produce high pollen sterility in autotetraploid rice hybrids that could be overcome by double pollen fertility neutral genes (*Sa*^*n*^ and *Sb*^*n*^) [14, 15]. Consequently, how to create new tetraploid rice lines with normal fertility and to overcome the sterility of F_1_ hybrids is a key step to use the tetraploid rice. After years of unremitting efforts, our research group has successfully developed a new “autotetraploid rice lines” by selective breeding and crossing for successive generations. The new “autotetraploid rice” displayed high fertility (> 80%) and high heterosis when crossed with other autotetraploid rice lines having low fertility [16–18]. Moreover, F_2_ and F_3_ populations also displayed high fertility and stable morphological traits like neo-*Arabidopsis* [16, 18, 19]. The new “autotetraploid rice” wasn’t an allotetraploid rice; however, its chromosome behavior was nearly normal, which contributed to high fertility and harbors specific DNA mutations that were different from autotetraploid rice. Therefore, we defined new “autotetraploid rice” as neo-tetraploid rice [16].

RNA-seq-based transcriptome data enables to understand the role of differentially expressed genes associated with abiotic stress and pollen development in rice [20, 21]. Using RNA-Seq, significant progress has been made in understanding the expression patterns of genes during pollen development between diploid and autotetraploid rice over the last few years [12, 13, 22]. Furthermore, RNA-seq has also been widely used in the study of genetic variations in plants. The complexity of gene expression profiles associated with heterosis in different tissues had been revealed in various plants, such as rice [16, 17, 23, 24], tobacco [25], wheat [26, 27], rapeseed [28], and maize [29]. Transcriptome diversity between hybrid rice and their parents was analysed in various tissues during different development stages and specific differentially expressed genes related with heterosis were identified in rice [16, 17, 23, 30–32].

Length of panicle, as a part of rice panicle architecture, is an important agronomic trait of rice, which significantly affects the rice yield. Many genes related to diploid rice panicle architecture have been identified or cloned, such as *OsSPL18* [33], *IPA1* /*OsSPL14* [34], *Ghd7* [35], *DEP1* [36], *LP* [37], *Gn1a* [38], *GW8*/*OsSPL16* [39], and *SPL* [40]. *OsSPL18* had the same gene structure and expression pattern as that of *GW8*/*OsSPL16*, and it regulates the expression level of *DEP1* [33]. *IPA1*/*OsSPL14* is regulated by *OsmiR156* [34], and *DST* enhanced grain production by controlling the expression patterns of *Gn1a*/*OsCKX2* [41]. *FRIZZLE PANICLE* (*FZP*) is a major negative regulator of *RFL*/*APO2* and determined the transition from panicle branching to spikelet formation [42]. However, little information is available about long panicle in neo-tetraploid and autotetraploid rice.

In this study, we reported the breeding procedure of a neo-tetraploid rice with long panicles, harboring wide-compatibility genes, which could overcome F_1_ sterility. This study was planned to evaluate the heterosis of neo-tetraploid rice crossed with different autotetraploid rice lines, and to analyse molecular aspects of heterosis using RNA-seq-based transcriptome and re-sequencing analysis. Moreover, the expression patterns of important genes were validated by qRT-PCR. Our study will provide new germplasm for polyploid rice breeding and help us to understand the variations in gene expressions associated with heterosis and long panicle between F_1_ hybrids and their parents.

## Results

### Development of neo-tetraploid rice with long panicle and its genotype at F_1_ pollen sterility loci

**Fig. 1:**
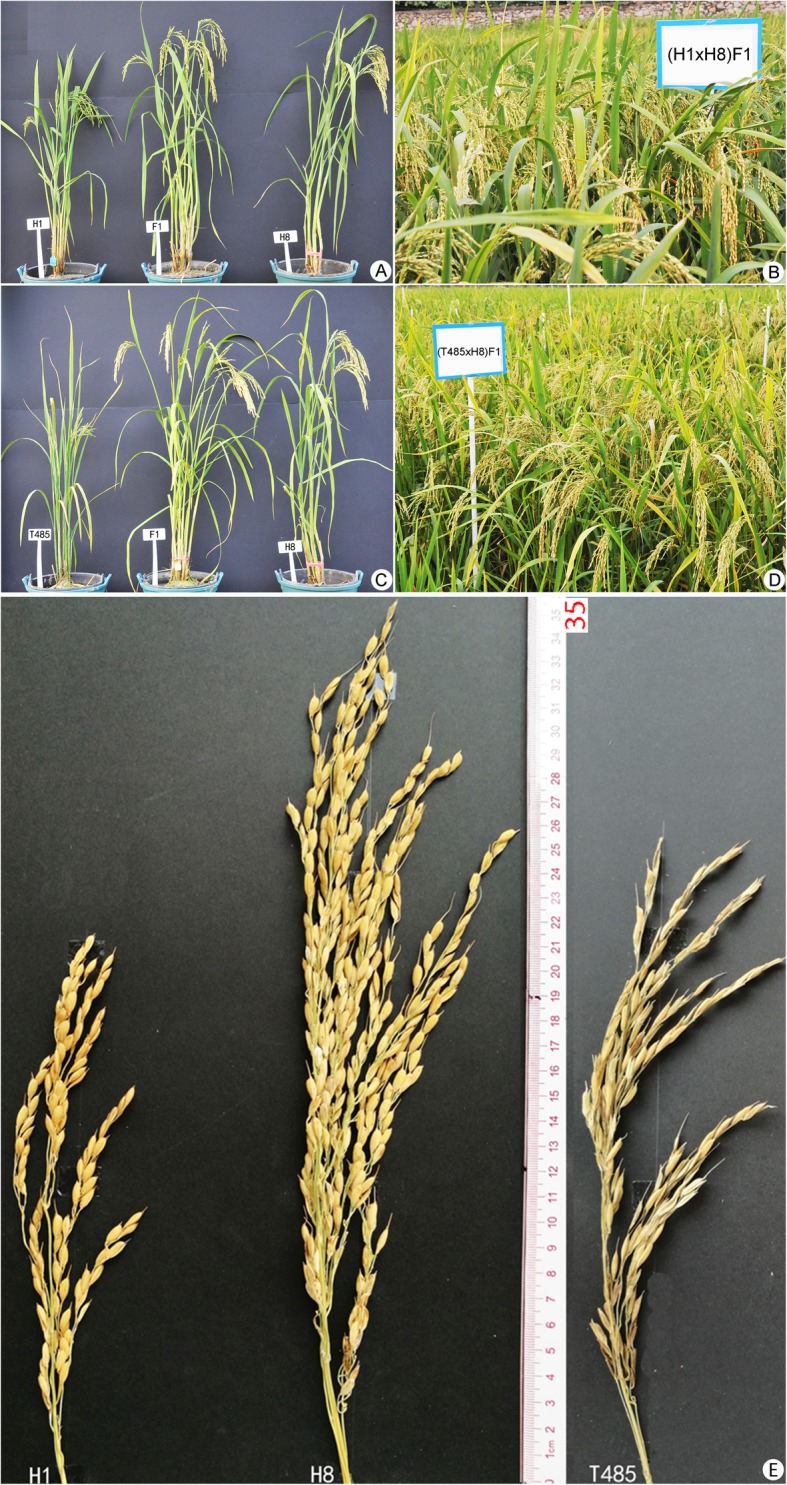
Morphological characteristics of the parents and two hybrids. **a**, **c**: Plant appearance of parents and two F_1_ hybrids (H1 × H8 and T485 × H8); **b**, **d**: Performance of the two hybrids in the field (maturity stage); **e**: Panicles of H1, H8 and T485. T485, H1and H8 indicate Huanghuazhan-4x, Huaduo 1 and Huaduo 8, respectively

In order to improve the panicle length of neo-tetraploid rice, an *indica* autotetraploid rice line, Linglun-4x, with an average of 27.65 cm long panicle and harboring *S*_*5*_^*n*^, was used as maternal to cross with a *japonica* autotetraploid rice line, L202-4x, with an average panicle length of 24.25 cm and harboring *S*_*5*_^*n*^, in 2004. The F_1_ generation was continuously self-crossed until F_16_, and one line with 30 cm long panicles and 60% seed set was developed in 2013 (F_17_). We planted that line continuously and developed a new line with long panicles from F_18_ to F_20_ generations. A neo-tetraploid rice line with 35 cm long main panicle was developed and named as “Huaduo 8” in 2015 (Figure S1a; Figure S1c). Huaduo 8 (H8) exhibited long panicles with narrowly distributed primary and secondary branches (Fig. 1; Figure S1b), and displayed high stability in F_3_ to F_5_ generations, like neo-tetraploid *Arabidopsis* (Figure S1c).

**Fig. 2:**
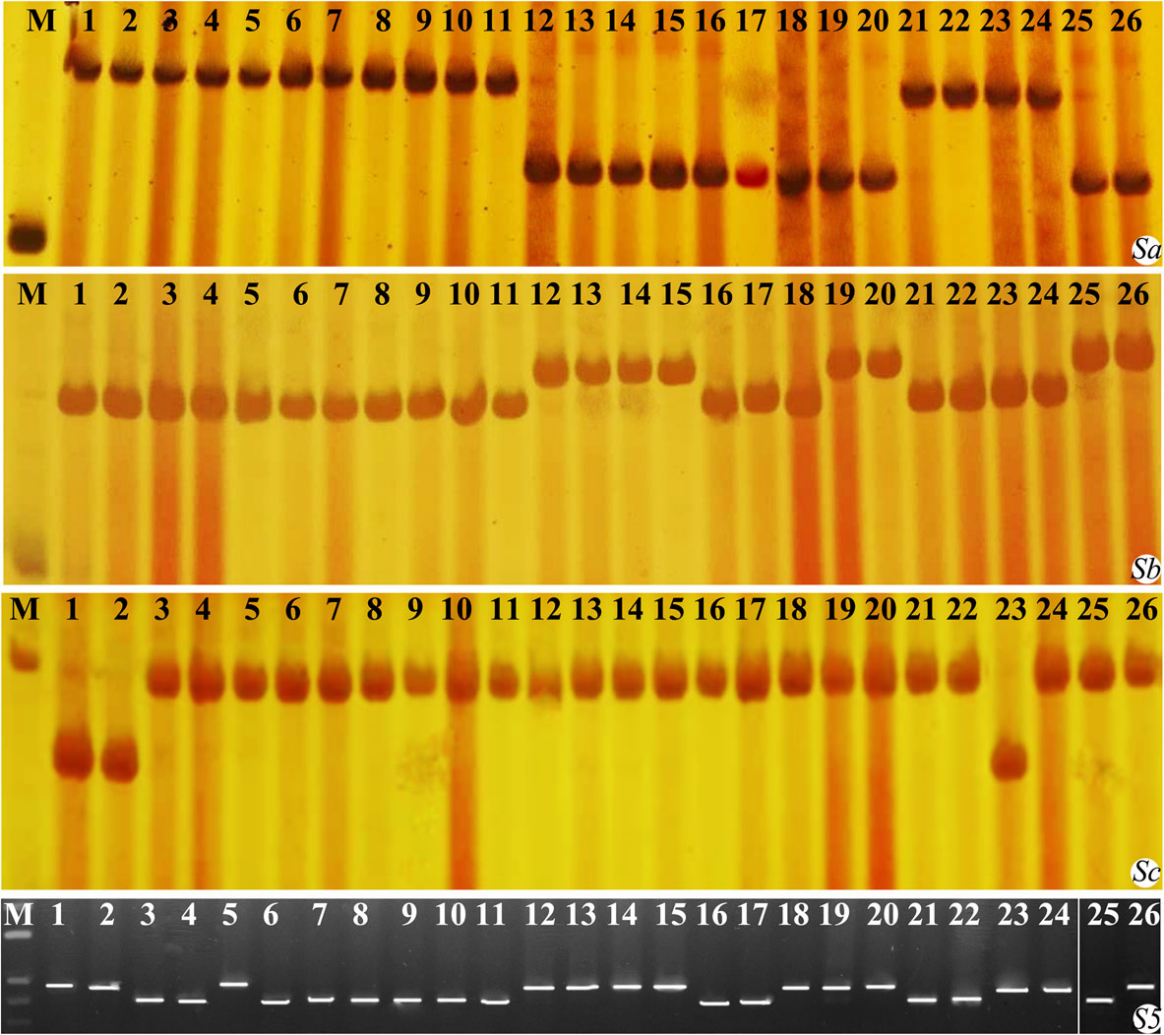
Genotypes of parental lines at pollen sterility loci (*Sa*, *Sb* and *Sc*) and *S5* locus detected by molecular markers. First image indicates the genotypes at *Sa* locus, followed by (upper to lower) *Sb*, *Sc* and *S5* locus. M indicates DNA ladder, lanes 1–4 indicate control cultivars (typical *indica* and *japonica* cultivars), while lanes 5–26 indicate neo-tetraploid/autotetraploid rice lines (see the name of materials in Additional file 2: Table S1). Lanes 25 and 26 in S5 were taken from another gel

Since our previous study revealed that interactions at F_1_ pollen sterility loci, *Sa*, *Sb* and *Sc* play key role in autotetraploid rice hybrid sterility, the genotypes of H8 and other parental lines at the *S-a*, *S-b*, and *S-c* loci were detected using closely linked molecular makers. The results revealed that H8 and other parental lines had different alleles at three loci, suggesting that hybrids exhibited different pollen sterility loci interactions at these loci. Moreover, the genotype of all materials at *S*_*5*_ locus was also detected using *S*_*5*_ functional molecular marker and the results indicated that H8 and 13 other parents contained *S*_*5*_^*n*^ gene (Fig. 2; Additional file 1: Figure S2; Additional file 2: Table S1). These results suggested that H8 is a wide-compatibility germplasm of neo-tetraploid rice.

### Hybrid vigor analysis of the neo-tetraploid rice with long panicle

Here, a total of 14 hybrids, including nine hybrids that were developed by crossing H8 with autotetraploid rice lines (i.e. four *japonica* and five *indica* lines), and five from crossing with other neo-tetraploid rice lines with high fertility (70.63%) (Additional file 2: Table S2). For panicle length, six hybrids showed positive significant high parent heterosis (HPH), and T49 × H8 depicted the highest HPH. Notably, all the hybrids displayed long panicles, and T49 × H8 exhibited the largest panicle length i.e. 37.61 cm (Additional file 1: Figure S1b and S1c; Additional file 2: Table S3). Notably, yield and yield components of these hybrids displayed significant improvement, especially seed setting, grain yield per plant, and filled grains per plant. Of these traits, the mean value of seed setting was higher than 77.04%, grain yield per plant was higher than 26.50 g, and filled grains per plant was higher than 650 in all hybrids (Additional file 2: Table S3).

All the hybrids exhibited significant positive mid parent heterosis (MPH) for all traits except the number of panicles per plant, and significant positive HPH for number of grains per plant and 1000-grain weight in both seasons. Three F_1_ hybrids displayed good performance for important agronomic traits, i.e. T45 × H8 showed significant positive HPH and MPH for all traits except HPH for 1000-grain weight, T419 × H8 displayed significant positive HPH and MPH for all traits except the number of panicles per plant, and T49 × H8 exhibited significant positive HPH for all traits except the number of panicles per plant and seed setting, and also yielded the highest HPH and MPH for important traits, including panicle length, filled grains per plant and grain yield per plant (Additional file 2: Table S2). The yield-related traits, such as seed setting, grain yield per plant, and filled grains per plant improved significantly in the hybrids produced by crossing H8 with neo-tetraploid rice compared to the hybrids developed by crossing H8 with autotetraploid rice (Additional file 2: Table S3).

Among the 14 hybrids, we selected two hybrids, T485 × H8 and H1 × H8, to analyze F_1_ yield-related heterosis by transcriptome analysis. T485 is an autotetraploid rice line with low fertility (33.35%) and H1 is a neo-tetraploid rice line with high fertility (65.3%). The two hybrids showed high positive MPH and HPH for important yield-related traits, including seed setting, grain yield per plant, 1000-grain weight, and number of filled grains per plant. The yield of two hybrids was significantly higher than their parents (Additional file 2: Table S3). Moreover, the seed setting was high in F_2_ of H1 × H8, with an average of 71.35%. Interestingly, H1 × H8 showed better performance than T485 × H8 in all traits except panicle length (Table 1).

**Table 1:** Agronomic traits of F_1_ hybrids and their parents during two seasons

Traits	Parents (Mean ± SD)			F_1_ hybrids (Mean ± SD)	
	T485	H1	H8	T485 × H8	H1 × H8
Plant height (cm)	90.78 ± 0.25	101.39 ± 1.63	137.28 ± 3.60	132.28 ± 2.49	144.53 ± 1.11
No. of panicles per plant	4.78 ± 0.54	5.45 ± 0.98	3.11 ± 0.42	3.77 ± 0.31	4.67 ± 0.40
Panicle length (cm)	25.28 ± 0.87	20.72 ± 0.55	28.65 ± 0.26	28.44 ± 1.12	28.44 ± 1.13
Filled grains per plant	153.39 ± 40.13	252.73 ± 63.35	308.72 ± 29.09	505.33 ± 56.11	589.10 ± 42.79
Grain yield per plant (g)	5.14 ± 1.16	8.88 ± 2.13	10.89 ± 0.75	18.72 ± 1.94	23.18 ± 1.50
1000-grain weight (g)	34.68 ± 2.98	35.51 ± 0.22	35.35 ± 1.15	37.21 ± 0.71	39.55 ± 0.62
Seed setting (%)	33.35 ± 4.53	65.30 ± 3.65	55.82 ± 6.19	75.16 ± 2.25	77.04 ± 3.80

T485, H1and H8 indicate Huanghuazhan-4x, Huaduo 1 and Huaduo 8, respectively

### Transcriptome analysis of F_1_ hybrids and their parents

More than 5464 million clean reads were obtained from the two F_1_s, including T485 × H8 and H1 × H8, and their parents by RNA-seq of eight tissues (Additional file 2: Table S4a, b). We aligned these clean reads against the Nipponbare reference genome (MSU 7.0), and obtained an average of 95.11% annotated transcripts of the reference genome and 66.23% unique mapped reads. The correlation coefficient was more than 0.8 that displayed a significant correlation between three biological replicates of transcriptome data in the hybrids and their parents. We used eight pairs of primers to validate the expression levels of 32 samples by qRT-PCR, and the results demonstrated that the differential expression levels of eight genes were consistent with the transcriptome data (Additional file 1: Figure S3). Hierarchical cluster analysis revealed that most of the differentially expressed genes in F_1_ were more close to those in H1 (Additional file 1: Figure S4), suggesting that H1 contributed greatly to the high fertility and yield-related traits in the hybrid compared to other parent, T485.

### Detection of differentially expressed genes (DEGs) from different tissues and gene ontology (GO) enrichment analysis

A total of 150,377 DEGs, including 76,926 from H1 × H8 and 73,451 from T485 × H8, were detected from eight tissues, i.e. flag leaf, leaf sheath, main panicle axis and spikelet of two hybrids and their parents before flowering and 5 days after flowering, respectively. The DEGs were ranged from 857 to 8489 between two parents and F_1_ compared to its parents (Additional file 2: Table S5, S6).

The specific DEGs that were uniquely associated with F_1_ hybrid compared to parents (DEGFu-sp) and could elaborate the phenotypic variations between F_1_ hybrid and its parents [16, 23]; so we focused on the DEGFu-sp to detect the genes related to heterosis. In total, 24,045 DEGFu-sp genes were identified in the two hybrids. Among DEGFu-sp, 4013, 7050, 6787 and 6195 were specifically associated with flag leaf, leaf sheath, main panicle axis and spikelet in the two hybrids, respectively (Table 2; Additional file 2: Table S7).

**Table 2:** Differentially expressed genes (DEGs) in eight tissues of two hybrids and their parents

F_1_ Hybrids	Tissues	DEG2P	DEGP1	DEGP2	DEGFu-sp^a^
H1 × H8	0-L	6257	1465	4874	1162
	0-S	4716	1342	3730	1408
	0-P	2409	1534	2205	1548
	0-Z	3615	995	2745	1151
	5-L	2120	1131	857	521
	5-S	4503	3813	3972	3020
	5-P	8489	984	6049	1137
	5-Z	3602	1665	3854	1903
	Total	35,711	12,929	28,286	11,850
T485 × H8	0-L	6374	5082	1785	1568
	0-S	6309	4131	1485	1232
	0-P	3173	1539	1696	961
	0-Z	4872	1849	3707	1591
	5-L	2471	1506	1046	762
	5-S	3328	1358	2605	1390
	5-P	3882	2902	4320	3141
	5-Z	3364	1603	3064	1550
	Total	33,773	19,970	19,708	12,195

T485, H1and H8 indicate Huanghuazhan-4x, Huaduo 1 and Huaduo 8, respectively

0-L, Flag Leaf; 0-S, Leaf sheath; 0-P, Spikelet; 0-Z, Main panicle axis before flowering; 5-L, Flag leaf; 5-S, Leaf sheath; 5-P, Spikelet; 5-Z, Main panicle axis 5 days after flowering; DEGFu-sp^a^: Differentially expressed genes uniquely belonging to F_1_ compared to parents (DEGFu-sp) associated with flag leaf, leaf sheath, spikelet and main panicle axis in the two hybrids before and 5 days after flowering

Gene ontology (GO) enrichment analysis revealed remarkable differences in the prominent functional categorization of genes in the eight tissues before and 5 days after flowering (Additional file 1: Figure S5; Additional File 2: Table S8a-c;). We identified 151 prominent terms in the biological process category, 61 in the molecular function category and 55 prominent terms in the cellular component category associated with DEGFu-sp to eight tissues (Table 3). Since two F_1_ hybrids (i.e. T485 × H8 and H1 × H8) were developed using neo-tetraploid rice line with long panicle to analyze the yield-related heterosis, we focused on the specific common genes from DEGFu-sp and their prominent functional categories. A total of 280 DEGFu-sp with specific common elements, in which 20 prominent GO categories, such as GO:0045449 (regulation of transcription), GO:0060255 (regulation of macromolecule metabolic process), and GO:0003700 (transcription factor activity), were detected in the leaf sheath of both hybrids that was collected 5 days after flowering. In total, 196 DEGFu-sp with specific common elements, in which 21 prominent GO categories, including GO: 0043687 (post-translational protein modification), GO: 0006464 (protein modification process), GO: 0043412 (macromolecule modification), were identified in main panicle axis of both hybrids 5 days after flowering (Additional file 2: Table S8d).

**Table 3:** The prominent functional categorization of differentially expressed genes uniquely belonging to F_1_ compared to parents (DEGFu-sp) associated with different plant tissues

F_1_ hybrids	0-L	0-S	0-P	0-Z	5-L	5-S	5-P	5-Z	Total
H1 × H8	32	22	46	21	12	40	9	38	220
T485 × H8	67	12	24	65	47	41	83	32	371
Total	97	34	70	86	59	81	92	70	591

T485, H1and H8 indicate Huanghuazhan-4x, Huaduo 1 and Huaduo 8, respectively

0-L, Flag Leaf; 0-S, Leaf sheath; 0-P, Spikelet; 0-Z, Main panicle axis before flowering; 5-L, Flag leaf; 5-S, Leaf sheath; 5-P, Spikelet; 5-Z, Main panicle axis 5 days after flowering

KEGG analysis revealed a total of 19 and 44 pathways associated with the DEGFu-sp in the two hybrids. Overall, 15 pathways were common in both hybrids, including STARCH and SUCROSE METABOLISM, PHOTOSYNTHESIS, PLANT-PATHOGEN INTERACTION, and CARBON FIXATION IN PHOTOSYNTHETIC ORGANISMS (Additional file 2: Table S8e).

### Functional analysis of DEGs associated with heterosis in different tissues

Since the heterosis was involved in all biological traits of rice hybrids, and the most important traits were grain yield and resistance, we analyzed the genes associated with kinase and synthase, including protein kinase, starch biosynthesis and their related genes [23, 43]. Of the DEGFu-sp to the two hybrids, 279 and 89 genes were involved in kinase and synthase, respectively (Additional file 3: Table S9a, S9b; Table 10Se and 10Sf). Of these 279 genes associated with kinase, 15 genes were annotated as receptor protein kinase and 164 protein kinase, such as S-locus lectin protein kinase family protein, and OsWAK38-OsWAK receptor-like protein kinase (Additional file 3: Table S9a). Among 89 genes annotated as synthase, 16 genes were encoded as cellulose synthase, 10 for ATP synthase and 7 for phosphate synthase (Additional file 3: Table S9b). Moreover, 22 genes encoding NBS-LRR disease resistance protein or LRR receptor-like protein kinase and 76 transcription factors (TFs) were detected in the DEGFu-sp to both hybrids (Additional file 3: Table S9c, 9d). Of these, 21 genes were different from super hybrid diploid rice LYP9 [23] and 11 were the same as those in F_1_ hybrid (T449 × H1) [17], and 71 TFs were different from super hybrid diploid rice LYP9 [23] and 29 were the same as those in F_1_ hybrid (T449 × H1) [17]. Moreover, we compared with the data of cloned genes (https://funricegenes.github.io/), and a total of 714 common cloned genes were detected in the DEGFu-sp genes of the two hybrids (Additional file 3: Table S9e; Table 10Se and 10Sf)*.* These genes could be divided into four groups, including morphological traits-related genes (at least 38 genes), physiological traits-related genes (at least 33 genes), resistance or tolerance-related genes (at least 66 genes) (Additional file 3: Table S9e) and other genes (577 genes). Among the morphological traits-related genes, some important traits related genes, such as grain number (*Gn1a*), and semi-dwarf (*sd1*) were detected. Four genes associated with eating quality were also identified in the physiological traits-related genes. Of the resistance or tolerance-related genes, 5 bacterial blight resistance genes, 9 blast resistance, 5 cold tolerance, 10 drought tolerance, and 9 salinity tolerance genes were identified (Additional file 3: Table S9e). Then, we compared the DEGFu-sp genes of the two hybrids with the genes associated with heterosis in diploid hybrid rice [44], and detected important genes, including *NAL1*, *qGW8*, *OsGA20ox1*, *Ghd8*, *GW6a*, *LP1*, and *EUI1*, *i-sd-1(t)* (elongated uppermost internode), *sd1*, *OsGA20ox2* and *qSD1–2* (dee-geo-woo-gen dwarf), *Hd3a* and *FT* (heading date), *DEP1, DN1, qPE9–1* and *qNGR9* (DENSE PANICLE), and *OsWRKY71*.

### Mapping of DEGFu-sp in known quantitative trait loci (QTLs)

**Fig. 3:**
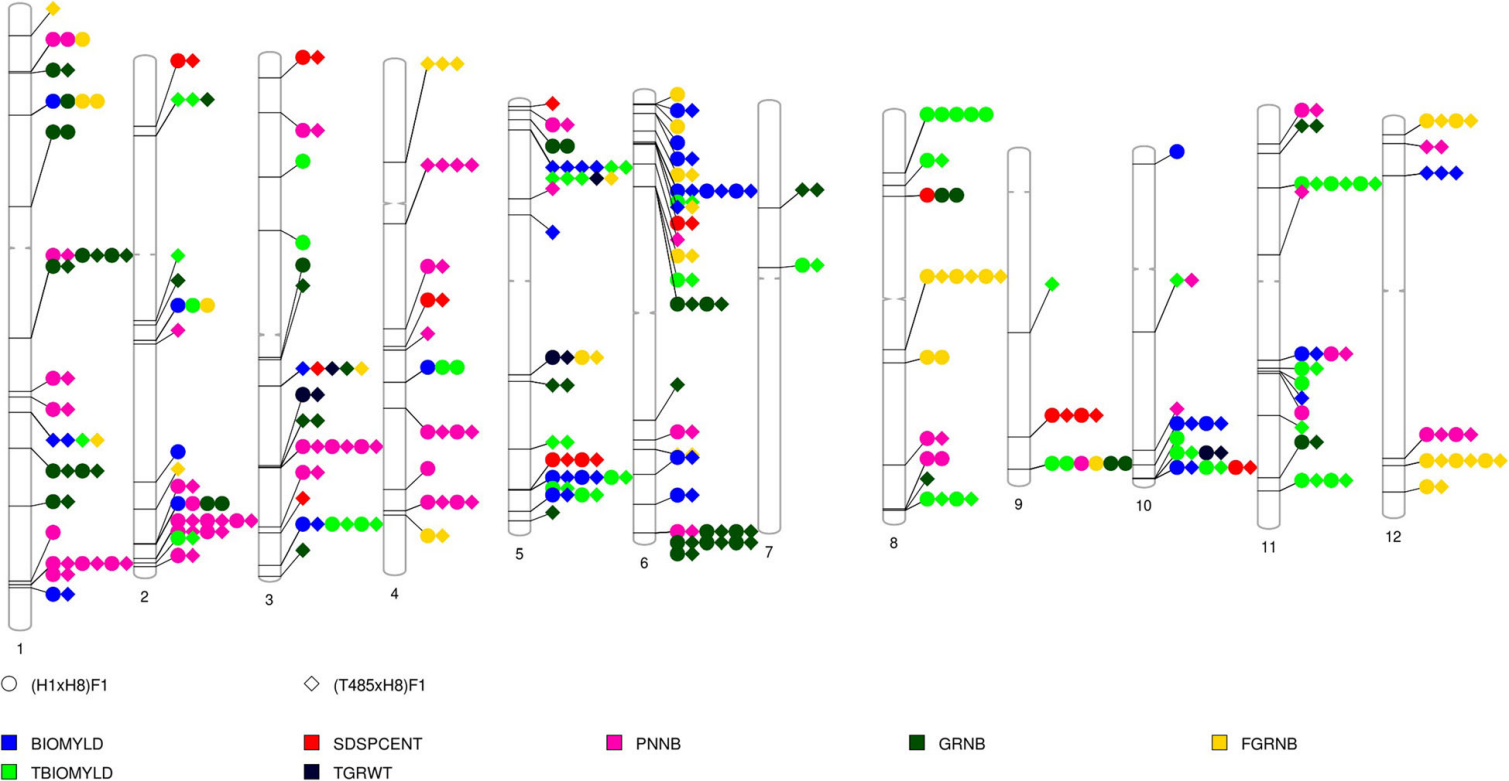
Distribution of the DEGFu-sp mapped onto known yield-related QTLs. The DEGFu-sp were mapped onto the seven rice yield-related QTLs, including total biomass yield (TBIOMYLD), biomass yield (BIOMYLD), filled grain number (FGRNB), grain number (GRNB), panicle number (PNNB), seed set percent (SDSPCENT) and 1000-grain weight (TGRWT)

The DEGFu-sp to two hybrids were used to map 8216 rice QTLs containing 236 traits (items) in the rice Gramene database (http://qtaro.abr.affrc.go.jp/), and the yield-related QTLs were analyzed. A total of 2925 QTLs intervals, with an average of 1~100 genes per interval, were identified, and 975 were common in both hybrids (Additional file 3: Table S10a,10b). Among the DEGFu-sp-related common QTLs, 109 rice yield-related QTLs associated with seven traits, including total biomass yield, biomass yield, number of filled grains, total of number grains, number of panicles, seed set and 1000-grain weight, were detected. Many QTLs are well characterized, including seed set (AQCB017, AQCB018, AQGH011, AQGH028, AQBK049, AQBK050, AQED063, CQAS35 and CQB14), 1000-grain weight (AQAI071, AQAI076 and CQB16), and number of filled grains (AQCY010, AQCY030, AQCY041, AQCY055, AQCY061, AQCY089, AQCY100, AQCY109, AQCY111 and AQCY115) (Fig. 3; Additional file 3: Table S10b, S10c). A total of 15, 9, 10, 7, 11, 22, 1, 7, 2, 7, 10 and 8 common QTLs were detected on chromosome 1, 2, 3, 4, 5, 6, 8, 9, 10, 11 and 12, respectively (Fig. 3). We checked the functional annotations of the common QTL-related DEGFu-sp, and some of them showed potential association between DEGFu-sp and QTLs, for example relationship between *JMJ706* (*LOC_Os10g42690*, H3K9 demethylase), *OsClpB-m* (*LOC_Os02g08490*, ATPase) and AQCB018, AQED063 for seed set, respectively. *Alpha amylase* (*LOC_Os05g32710*) and CQB16 for 1000-grain weight, and *OsLpa1*(*LOC_Os02g57400*, low phytic acid 1) and AQGI200 for total biomass yield.

Moreover, we compared the DEGFu-sp-related QTLs data with two F_1_ hybrids of neo-tetraploid rice T449 × H1 [17] and T452 × H3 [16]. A total of 48 QTLs were common among the present and previous studies, including two were associated with 1000-grain weight (AQAI071 and CQB16), three with seed set (AQGH011, AQGH028 and CQB14), and 13 with number of panicles (Additional file 1: Figure S6; Additional file 3: Table S10d). Then, we compared the DEGFu-sp (DGHPU)-related QTLs data with super hybrid diploid rice LYP9 [23], and detected 57 different QTLs, including four associated with seed set (AQGH011, AQGH028, AQCB017 and AQED063), 15 with biomass yield, 14 with number of panicles and 24 with number of grains (Additional file 3: Table S10c).

### Non-additive gene expressions in F_1_ hybrids

The DEGFu-sp could be divided into two types, i.e. additive and non-additive genes, which contributed by each allele from both parents and that deviates from the mid-parent value, respectively [16, 23]. A total of 1393 non-additive DEGs, 766 up-regulated and 627 down-regulated, were detected in both hybrids, including 166, 362, 516 and 349 in leaf, sheath, panicle branches and main axis of panicles before and 5 days after flowering, respectively (Table 4; Additional file 4: Table S11a,b). We compared all the non-additive DEGs with the cloned genes data of rice, and found 13 genes with known functions in rice. Among 766 non-additive up-regulated genes, four genes i.e. *LOC_Os05g51610*, *LOC_Os12g05709*, *LOC_Os09g11490* and *LOC_Os11g28184* were found be common in leaf before fertilization in both hybrids. Twenty-nine genes were identified in main panicle axis before fertilization, 21 in spikelet after fertilization, one in flag leaf after fertilization, ten and five common genes in leaf sheath and main panicle axis were detected after fertilization in both hybrids, respectively (Additional file 4: Table S11c, d).

**Table 4:** Non-additive differentially expressed genes (NDEGs) and number of differentially expressed genes uniquely associated with F_1_ (DEGFu-sp)

F_1_ Hybrids	0-L	0-S	0-P	0-Z	5-L	5-S	5-P	5-Z
H1 × H8	79	55	104	109	24	179	180	112
up	62	40	48	86	20	86	100	47
down	17	15	56	23	4	93	80	65
T485 × H8	48	61	39	77	15	67	193	51
up	21	41	32	58	7	32	63	23
down	27	20	7	19	8	35	130	28
%	4.65	4.39	5.70	6.78	3.04	5.58	6.38	4.72

0-L, Flag Leaf; 0-S, Leaf sheath; 0-P, Spikelet; 0-Z, Main panicle axis before flowering; 5-L, Flag leaf; 5-S, Leaf sheath; 5-P, Spikelet; 5-Z, Main panicle axis 5 days after flowering

%: indicate the percent ratio of NDEGs to DEGFu-sp

Notably, H1 × H8 (F_1_ hybrid) showed better performance than T485 × H8, so we compared the non-additive DEGs overlapping with the cloned genes in both hybrids, and detected seven (*LOC_Os02g12350*, *LOC_Os03g17350*, *LOC_Os10g42750*, *LOC_Os08g41940*, *LOC_Os10g03400*, *LOC_Os04g46940* and *LOC_Os04g57830*) and two genes (*LOC_Os03g06654* and *LOC_Os03g43990*) specifically associated with H1 × H8 and T485 × H8, respectively. Moreover, two genes, i.e. *LOC_Os12g18360* (*Pi-ta*) and *LOC_Os03g51970* (growth regulating factor), which regulated by *Osa-miR396*, were found to be common in two hybrids (Additional file 4: Table S11c, d).

### Genome-wide DNA variations in the neo-tetraploid rice with large panicle

A total of 2,082,268 SNPs and InDels were detected in the neo-tetraploid rice with large panicle (H8) compared with a neo-tetraploid rice with short panicle (H1), in which 82,319 homologous mutants associated with 15,146 genes were detected in H8 compared to H1. We compared all the mutant genes with the functionally characterized genes of rice, and 257 genes were found with known functions in rice (Additional file 4: Table S12a). In total, 2,953,864 SNPs and InDels were detected in the neo-tetraploid rice with large panicle (H8) compared to the autotetraploid rice (T485). A total of 131,112 homologous mutants associated with 24,777 genes were detected in H8 compared with T485, and 191 genes had known functions in rice (Additional file 4: Table S12b). A total of 59 specific mutant genes were detected in H8, including large panicle gene *LP* (*LOC_Os02g15950*), *LPA1* (*LOC_Os03g13400*), *dwarf 61* (*LOC_Os01g52050*), *dwarf 10* (*LOC_Os01g54270*), *cellulose synthase catalytic subunit A4* (*LOC_Os01g54620*, *OsCesA4*), and *LAX PANICLE* (*LOC_Os01g61480*) (Additional file 4: Table S12c). Moreover, H8 had missense variants in *Hd1* compared to H1, while H1 exhibited a missense variant in *NAL1* compared to Nipponbare reference genome. T485 had a missense variant in *OsGA20ox1* compared to H8. Twelve specific mutated genes related to rice panicle architecture were also identified in H8, including *OsClpC2*, *OsClpC3*, *LPL3*, *OsGLP8–12*, *SP1* (*OsNPF4.1*), *OsERF71*, *OsHSP1*, *OsHSP17.7*, *spl5* (*SF3b3*; *0sSL5*), *SPL35* and *SPL3* (*OsEDR1*; *OsACDR1*; *OsMAPKKK1*).

In order to check the DNA variations in LP gene, we analyzed the re-sequencing data of 132 different rice lines, including 67 neo-tetraploid (including sister lines), 36 autotetraploid and 29 diploid rice lines. The results displayed that the DNA variations in LP gene of H8 were different from most of the other neo-tetraploid, autotetraploid and diploid rice lines (Additional file 1: Figure S7). The DNA sequences of LP gene in three parents, H8, H1 and T485, were validated by using Sanger sequencing, and the results were consistent with the data of re-sequencing.

Since many DNA sequences variations were detected among three parents, H1, T485 and H8, we focused on the gene variations in DEGFu-sp. A total of 33 genes showed DNA sequence variations (Additional file 4: Table S13a), including 3TE, 13 resistance or tolerance genes, 10 and 19 genes were associated with physiological and morphological traits, respectively (Additional file 4: Table S13b).

### Functional analysis of DEGs associated with panicle length and grain number in neo-tetraploid rice with large panicle

**Fig. 4:**
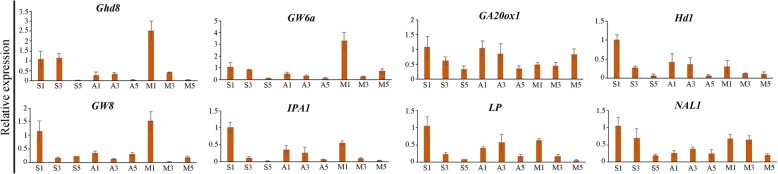
Expression levels of eight genes associated with heterosis in F_1_ hybrids. S1: 1 cm panicle length of F_1_ hybrid (H1 × H8); S3: 5 cm panicle length of hybrid F_1_ (H1 × H8); S5: 15 cm panicle length of F_1_ hybrid (H1 × H8); A1: 1 cm panicle length of H8; A3: 5 cm panicle length of H8; A5: 15 cm panicle length of H8; M1: 1 cm panicle length of H1; M3: 5 cm panicle length of H1; M5: 15 cm panicle length of H1. T485, H1and H8 indicate Huanghuazhan-4x, Huaduo 1 and Huaduo 8, respectively

**Fig. 5:**
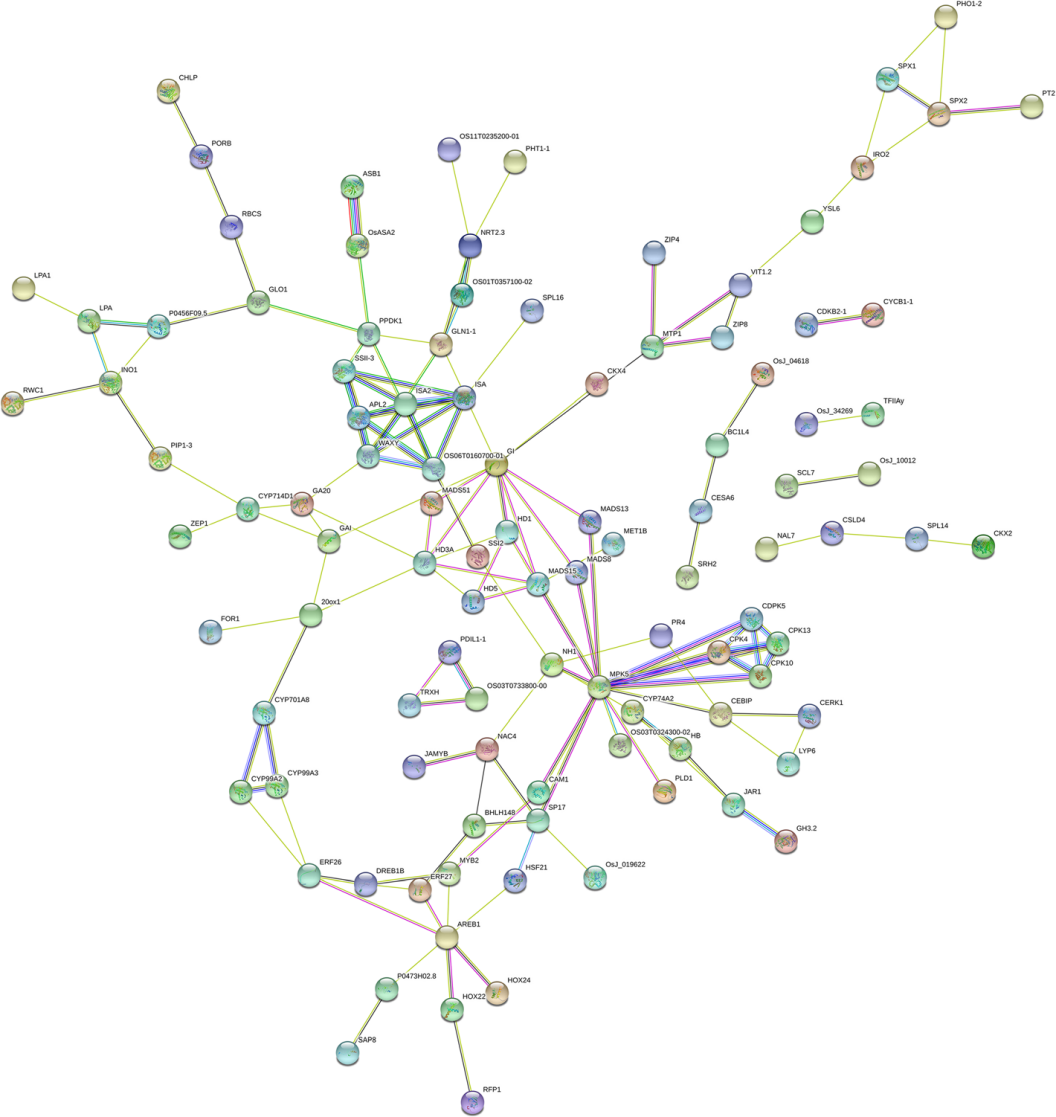
Predicted protein-protein interaction network of cloned and eight genes associated with heterosis in neo-tetraploid rice

In order to check the DEGs and important genes associated with panicle length and number of grains per panicle in neo-tetraploid rice with large panicle, RNA-seq was employed to analyze global gene expressions in very young panicles of H1 × H8 vs H1 (short panicle), H8 vs H1, T485 × H8 vs T485, and H8 vs T485. A total of eight up-regulated genes associated with panicle were detected in young panicles of the two hybrids compared with their parents, including *OsMADS3*, *OsMADS6* (*mfo1*), *MADS58*, *Gn1a*, *OsClpC2*, *OsLG1*, *MULTI-FLORET SPIKELET1* and *LAX PANICLE*. The expression patterns of eight genes associated with length and grain number of panicle and heterosis, including *LP*, *Ghd8*, *NAL1*, *GW6a*, *IPA1*, *GA20ox1*, *Hd1* and *GW8*, were detected using qRT-PCR during different stages of panicle development. The results showed that the eight genes were found to be up-regulated in H1 × H8 vs H8 in very young stage of panicle development, and *LP* displayed up-regulation in H1 × H8 compared to both parents in young panicles (Fig. 4). Predicted protein-protein interaction analysis of above mentioned eight genes and 133 cloned genes displayed interaction with each other (Fig. 5; Additional file 4: Table S14). Moreover, the segregation ratio of panicle length of H1 × H8 was investigated in F_2_ generation, and the mean length of panicle was 24.61 cm, which was very close to the average length of two parents (24.69 cm). There were 12 plants with longer panicles than that of H8, and 19 with shorter panicles than that of H1, with the shortest 14 cm. The average seed setting was 71.51% in the F_2_ generation, suggesting that most of the plants maintained high seed set in next generation.

## Discussions

### The neo-tetraploid rice with long panicle is a wide-compatibility germplasm and many important genes were associated with high hybrid vigor

Hybrid sterility not only exists in intersubspecific diploid rice hybrids but also in autotetraploid rice hybrids, which caused by the allelic interaction of F_1_ sterility loci, including *Sa*, *Sb* and *Sc*, and their neutral alleles have the potential to overcome the hybrid sterility [14, 15, 17, 45, 46]. In the present study, the closely linked molecular markers associated with *Sa*, *Sb* and *Sc* loci were used to detect the genotypes of Huaduo 8 (H8) at three loci, which was similar to E249, harboring *Sa-n* and *Sb-n* at *Sa* and *Sb* loci, and similar to E25 and T45, which contained *Sc-n* at *Sc* locus. Meanwhile, 14 hybrids, including 10 hybrids generated by crossing H8 with *indica* autotetraploid rice and 4 with *japonica* tetraploid rice, displayed high seed setting and hybrid vigor like Huaduo 1 and Huaduo 3 [16, 17]. These results suggested that H8 contain neutral genes for pollen sterility loci. Moreover, the genotype at *S*_*5*_ locus, which causes embryo sac sterility in intersubspecific hybrids, was detected using *S*_*5*_ functional molecular marker and H8 harbored *S*_*5*_^*n*^ gene as well (neutral allele at *S*_*5*_ locus). Interestingly, almost all plants displayed high seed setting in F_2_ and next generations. Therefore, we inferred that H8 is a new wide-compatibility neo-tetraploid rice, which will be a valuable germplasm for polyploid rice breeding.

The neo-tetraploid rice with long panicle possess high hybrid vigor and many important genes associate with heterosis in diploid rice were also detected in the neo-tetraploid rice hybrids, including *OsGA20ox2*, *qSD1–2*, *Hd3a*, *DEP1,* and qGW8 [36]. The transcription factors (TFs), as important regulatory genes, affect the phenotypes of rice hybrids [23]. Interestingly, most of TFs with differential expression patterns in the two hybrids of neo-tetraploid rice were different from those in diploid rice hybrid [23]. Moreover, we also detected many DEGFu-sp associated with NBS-LRR disease resistance protein, and most of them were also different from those in diploid rice hybrid [23]. Non-additive genes have pronounced effects on the plant phenotypes, and had been detected in rice or autotetraploid rice hybrids [16, 17, 23]. Here, a total of 1393 non-additive DEGs were also identified. Kinase and synthase, such as protein kinase and starch synthase, played important role in heterosis performance [23, 43]. We found many genes involved in kinase and synthase, and the KEGG analysis results revealed an important pathway, i.e. STARCH AND SUCROSE METABOLISM, which was consistent with the results of genes detection. These results showed that many important genes were involved in the high hybrid vigor of neo-tetraploid rice and it’s a very useful germplasm to decipher the underlying mechanism of hybrid vigor in polyploid rice.

### Many yield-related QTLs contributed to high heterosis in neo-tetraploid rice

The molecular mechanisms underlying heterosis is very complicated in rice, and many functional genes or QTLs had been detected in diploid rice hybrids. The gene expression profiles of super hybrid rice, LYP9, were investigated by gene-chip and found that the genes uniquely belonging to F_1_ hybrid compared to parents, and were enriched in the categories of energy metabolism and transport. Moreover, 2673 differentially expressed genes between hybrids and parents (DGHP) were mapped onto 3128 QTLs, and 53 were uniquely belonging to differentially expressed genes between hybrids and parents (DGHPU), which were classified into 9 categories and 209 traits in the rice genome [23]. Many QTLs, including *Hd3a*, *qGL3*, *OsmiR156h*, and *LAX2*, and non-additive genes related to the yield-related traits in two-line hybrid rice, were identified [47]. A total of 22 yield-related QTLs, including 15 grain number QTLs, 61000-grain weight QTLs and 1 yield per plant QTL that contributed largely to the grain number heterosis, were detected in a super-hybrid rice WFYT025 [48]. More than 90% of the DEGFu-sp genes detected during different development stages (anthers and flag leaves at meiosis stage, flag leaves, leaf sheath, anther and ovary at pre-flowering stage, and flag leaves, leaf sheath and grains at 3 days after flowering) were mapped onto 1019 yield-related QTLs and 26 traits present in the rice Gramene database [17].

In the present study, we detected 2925 QTLs intervals, with a frequency of 1~100 genes per interval, and 975 were the same in the two hybrids, including 109 rice yield-related QTLs. Among the QTLs, 50 QTLs were the same as detected in our previous studies in the two neo-tetraploid rice hybrids (Huajingxian 74-4x × Huaduo 3 and T449 × Huaduo 1) [16, 17], such as two for 1000-grain weight (AQAI071 and CQB16), and three for seed set (AQGH011, AQGH028 and CQB14). Interestingly, some important genes-related QTLs, such as *JMJ706*, *OsClpB-m*, *Alpha amylase* and *OsLpa1*, and 26 specific QTLs were also found in the two neo-tetraploid rice hybrids. We inferred that the molecular mechanism involved in heterosis is complicated in the neo-tetraploid rice hybrid. There are at least five genetic factors that affect the phenotypes and produce high heterosis in the neo-tetraploid rice hybrids, including major functional genes, QTLs, TFs, NBS-LRR and epigenetic-related genes.

### The up-regulation and mutant genes association with panicle length may involve in the long panicle of neo-tetraploid rice, H8

There is a large genetic variance for panicle length of *indica* and *japonica* in rice. In general, *japonica* has short panicle and dense spikelet compared to *indica*, which is characterized by longer panicles and sparse spikelet [49, 50]. Panicle length was controlled by both major and minor QTLs in rice [51], and more than 253 QTLs and genes for panicle length distributed on 12 chromosomes have been reported [52–54]. A major rice grain yield QTL, *DEP1*, was identified, which increased number of grains per panicle by enhancing meristematic activity to reduce length of the inflorescence internode [36]. The larger panicle mutant gene, *LP*, could significantly increase panicle size, and encode F-box protein that could interact with rice SKP1-like protein [37].

In the present study, we detected 8 genes, including *OsMADS3*, *osmads6* (*mfo1*), *mads58*, *Gn1a*, “*OsClpC2”*, *OsLG1*, *MULTI-FLORET SPIKELET1* and *LAX PANICLE*, involved in the panicle architecture, which displayed up-regulation expression in F_1_ compared to parents. Among these genes, *Gn1a* controlled the filled grains per panicle [38]; *OsLG1* regulated a closed panicle trait in rice [55]. Actually, H8 is a neo-tetraploid rice with large and closed panicle trait, and the up-regulated expression patterns of these two genes, *Gn1a* and *OsLG1*, play important role in its panicle development. Moreover, we also detected DNA variations in some large panicle related genes, such as large panicle gene, *LP*.

## Conclusion

Here, we developed a neo-tetraploid rice line, Huoduo8, with long panicles, which could overcome intersubspefic hybrid sterility of autopolyploid rice. Huaduo 8 produced high heterosis when crossed with different autotetraploid and neo-tetraploid rice lines. We detected a total of 368 DEGFu-sp genes associated with kinase and synthase and 975 DEGFu-sp-related QTLs in the two hybrids generated by Huaduo 8. A total of eight genes, including a mutant allele of *LP*, related to rice panicle architecture, exhibited up-regulated expression patterns in very young panicles. Our research provided a very useful germplasm for breeding of high yielding polyploid rice, and provided insight into molecular mechanisms associated with heterosis in neo-tetraploid rice. Overall, molecular regulatory mechanism of heterosis is very complicated in neo-tetraploid rice, and future studies should be focused on the functional characterization of the genes associated with heterosis.

## Methods

### Evaluation of heterosis in a neo-tetraploid rice with large panicle

The materials used in this study were comprised of nine autotetraploid and five neo-tetraploid rice lines, which were crossed with a neo-tetraploid rice line with long panicle, Huaduo 8 (H8), to develop 14 hybrids (Additional file 2: Table S1). All the parents and their F_1_s were planted under the natural conditions at the experimental farm of South China Agricultural University (SCAU), Guangzhou (23^o^ l6 N, 113^o^ 8E) in two growing seasons i.e. late season (20th July to 30th November, 2017) and early season (25th February to 15th July, 2018). All the lines, except L202-4x, Jackson-4x and Luxiang 97-4x were kindly provided by Prof. Yuanqing Li, used in this study are produced by our research group and is being used from last several years [5–7, 10, 16, 18]. The voucher specimen of polyploid rice lines has been deposited to our lab but not to any publicly available herbarium. We didn’t use wild plants in this study and according to national and local legislation, no specific permission was required to collect these plants.

A total of 15 plants from all F_1_ hybrids and their parents were harvested from the middle part of the field at maturity. Agronomic traits, including panicle length (PL, cm), plant height (PH, cm), effective number of panicles per plant (NP), filled grains per plant (FG/P), 1000-grain weight (GWT, g), grain yield per plant (GY, g), and seed setting (SS%) were selected and measured according to our previous study [16]. In order to detect the genotypes at F_1_ pollen sterility loci, *Sa*, *Sb* and *Sc*, and at *S5* embryo sac sterility locus, molecular markers were used. Here, closely linked molecular markers were employed to detect genotypes at known loci, including one SNP marker (G02–148) for *Sa* locus, two markers (A07–55 and A07–130) for *Sb* locus, one marker (P24–85.7) for *Sc* locus, and one marker (S5-t1) to detect neutral gene (*S*_*5*_^*n*^) at S5 locus (Additional file 2: Table S15) [45, 46].

We analyzed the data and estimated the heterosis of hybrids in three replications during both seasons. The single factor variance analysis of each trait was done by SPSS 16.0 using 0.05 and 0.01 significance levels. Mid-parent heterosis (MPH) and high-parent heterosis (HPH) were determined by the following formula: MPH = (F_1_-MP)/MP × 100%, and HPH = (F_1_-HP)/HP × 100%, where MP is the average performance of two parents, F_1_ is the performance of first generation (hybrid), and HP is the performance of the best parent.

### Genome-wide DNA variations in a neo-tetraploid rice with large panicle and other parents

Young leaves of H8, Huanghuazhan-4x (T485) and Huaduo1 (H1) were collected and stored at − 80 °C for DNA isolation. Genomic DNA was extracted from each young leaf tissue by using a modified CTAB method [56]. The task of whole-genome re-sequencing was done by Biomarker Technologies (Beijing, China) on Illumina HiSeq platform. The method was performed in accordance with the standard Illumina protocol as described previously [57].

The quality of sequencing raw reads was evaluated by FastQC software, and the filtered high quality reads were aligned to the Nipponbare reference genome (MSU7) using BWA software. Genomic variations (InDels and SNPs) were detected using GATK software (https://www.broadinstitute.org/gatk/guide/best-practices.php). The InDels and SNPs were annotated based on the GFF3 file of the reference genome using SnpEff software. According to the location of the polymorphisms, the genic SNPs and InDels were classified as CDS (coding sequences), UTR (untranslated regions) and introns. The SNPs in coding sequencing were classified as synonymous and non-synonymous.

### RNA extraction, cDNA library construction, and RNA-Seq

Eight tissues, including flag leaf, leaf sheath, main panicle axis (including rachis and all branches but not spikelet), and spikelet (not including anther) at flowering stage before fertilization and 5 days after flowering were collected from two F_1_ hybrids (T485 × H8 and H1 × H8) and their parents (Additional file 1: Figure S8; Additional file 2: Table S1;). Moreover, the young panicles with a length of less 0.5 cm were also collected from the two hybrids and their parents. All the samples were collected in three biological replications and kept at − 80 °C for RNA extraction.

The total RNA from each sample was extracted based on the manual instruction of the TRlzol Reagent (Life technologies, California, USA), and libraries were prepared as described previously [16]. The quality and quantity of all the samples were evaluated by a Nanodrop 1000 spectrophotometer and 1% agarose gel. An Agilent 2100 Bioanalyzer (Agilent Technologies, Inc., USA) was used to estimate the number and RNA integrity concentration. NEBNext Poly (A) mRNA magnetic extraction module was employed to extract the mRNA. The first- and second-strand cDNA were produced by breaking the purified and enriched mRNA into about 200 nt short RNA inserts. The end-repair/dA-tail and adaptor ligation was executed of double-stranded cDNA. AgencourtAMPure XP beads (Beckman Coulter, Inc.) were used to extract the appropriate fragments, and amplified by PCR. Then, an Illumina HiSeq™ 2500 sequencing platform was employed to sequence the cDNA libraries [16, 18].

The low quality reads were eliminated by perl script as described previously [16]. The filtered clean reads were mapped onto Nipponbare reference genome (IRGSP-1.0 pseudomolecule/MSU7) using Tophat2software and Bowtie2 [58]. The aligned data were further checked to eliminate potential duplicate reads. The Cufflinks software was used to assess gene expression patterns by Fragments Per Kilobase of transcript per Million fragments mapped (FPKM) [59].

Differentially expressed genes (DEGs) between F_1_s and their parents were evaluated by DESeq. Then, gene abundance differences between F_1_s and their parents were estimated based on the ratio of the FPKM values. To calculate the significant differences, the false discovery rate (FDR) control method was applied to detect the the *P*-value in various investigations. In the present study, the genes with FDR significance score < 0.01 and an absolute fold change value ≥2 was used for further analysis [16, 18].

Gene Ontology (GO) analysis was conducted to annotate the DEGs using the AgriGO tool (http://bioinfo.cau.edu.cn/agriGO/), and Pathway analysis was done by Plant GeneSet Enrichment Analysis Toolkit (http://structuralbiology.cau.edu.cn/ PlantGSEA/). Cluster analysis was performed using Cluster 3.0 software. Predicted protein-protein interaction analysis was performed using STRING software (http://www.string-db.org/). Venny software was employed to detect the overlapped differentially expressed genes in different samples and tissues (http://bioinfogp.cnb.csic.es/tools/venny/) [14].

### Mapping of DEGFu-sp to rice QTLs

Rice QTL data with physical positions on the MSU Rice Genome Annotation Project Release 6.1 were acquired from Gramene (ftp://ftp.gramene.org/pub/gramene/archives/qtl/) [60]. The DEGFu-sp were mapped onto the seven yield-related QTLs, including total biomass yield (TBIOMYLD), biomass yield (BIOMYLD), filled grain number (FGRNB), grain number (GRNB), panicle number (PNNB), seed set percent (SDSPCENT) and 1000-grain weight (TGRWT), using gene coordinates from the MSU Rice Genome Annotation Project (Additional file 4: Table S16) (http://qtaro.abr.affrc.go.jp/) [17, 23].

### qRT-PCR

A total of 30 DEGs were randomly selected from the transcriptome data of eight tissues to validate RNA-seq data by qRT-PCR with eight primers. Moreover, we designed another eight primers to observe the expression patterns of eight genes during development of panicle, including 1 cm, 5 cm and 15 cm long panicles (Additional file 4: Table S15). The Transcriptor First Strand cDNA Synthesis Kit (Roche) was used to obtain first-strand cDNA from approximately 1 μg of extracted RNA as described previously [16]. The components of each reaction was 10 μM of forward and reverse primers, 10 μL of universal SYBR Green supermix (Bio-RAD) and 2 μL of cDNA and the final volume of each qRT-PCR reaction was 20 μL. The PCR reaction program was denaturation for 30 s at 95 °C followed by 40 cycles of amplification (i.e. 95 °C for 5 s and 60 °C for 30 s). We utilized rice Actin gene as an internal control, and all the reactions were executed in triplicate. The 2^–ΔΔCT^ method was applied to estimate the relative expression patterns [61].

## Supplementary information

**Additional file 1: Figure S1a.** Breeding procedure of Huaduo 8. **Figure S1b** Panicles of Huaduo 1 (H1), Huaduo 8 (H8) and their F_1_ hybrid. **Figure S1c** Plant appearance of parents and F_1_ hybrid generated by crossing with Huaduo 8 (H8). **Figure S2** Original images of genotypes of parental lines at pollen sterility loci (*Sa*, *Sb* and *Sc*) and *S5* locus detected by molecular markers. **Figure S3** qRT-PCR validation for the quality of transcriptomic data. **Figure S4** Hierarchical clustering analysis of all expressed genes based on transcriptome data. **Figure S5** Prominent functional categories of genes in the eight tissues detected by Gene ontology enrichment analysis before and 5 days after flowering in two hybrids. **Figure S6** Distribution of the common seven yield-related QTLs in the two F_1_ hybrids, T449 × H1 [17] and T452 × H3 [16], compared to the DEGFu-sp-related QTLs detected in the present study. **Figure S7** Cluster analysis of the DNA variations in LP gene of Huaduo 8. **Figure S8** Samples used for RNA-seq analysis.

**Additional file 2: Table S1.** Origin and genotypes of autotetraploid and neo-tetraploid rice lines at pollen sterility loci, *Sa*, *Sb*, and *Sc*, and embryo sac fertility locus (*S5*), and their agronomic traits. **Table S2.** Means of mid-parent and high-parent heterosis in F_1_ during two seasons of 2017 and 2018. **Table S3.** Means of agronomic traits of F_1_ in two seasons of 2017 and 2018. **Table S4a**. Codes and names of the parents, hybrids, tissues and development stages. **Table S4b**. Summary of clean reads of transcriptome data from the two F_1_ hybrids (H1 × H8 and T485 × H8) and their parents. **Table S5** Differentially expressed genes associated with the F_1_ hybrids compared to parents in flag leaf, leaf sheath, spikelet and main panicle axis. **Table S6** Differentially expressed genes associated with parents in flag leaf, leaf sheath, spikelet and main panicle axis. **Table S7** Gene IDs of differentially expressed genes that uniquely associated with F_1_ compared to parents and specific (DEGFu-sp) to flag leaf, leaf sheath, spikelet and main panicle axis. **Table S8a** Prominent functional categorization of genes in the eight tissues before and 5 days after flowering in the F_1_ hybrid (H1 × H8) by Gene ontology (GO) enrichment analysis. **Table S8b** Prominent functional categorization of genes in the eight tissues before and 5 days after flowering in the F_1_ hybrid (T485 × H8) by Gene ontology (GO) enrichment analysis. **Table S8c** List of the functional categorization of genes in the hybrids by Gene ontology (GO) enrichment analysis. **Table S8d** Common prominent functional categorization of genes by Gene ontology (GO) enrichment analysis. **Table S8e** KEGG Pathway analysis of DEGFu-sp to the two hybrids.

**Additional file 3: Table S9a** The common genes associated with kinase in DEGFu-sp to both hybrids. **Table S9b** The common genes associated with synthase in DEGFu-sp to both hybrids. **Table S9c** The common genes associated with NBS-LRR disease resistance protein or LRR receptor-like protein kinase in DEGFu-sp to both hybrids. **Table S9d** The common genes associated with transcription factors in DEGFu-sp to both hybrids. **Table S9e** The known genes associated with important agronomic traits detected from the DEGFu-sp to both hybrids. **Table S10a** Names of all DEGFu-sp-related QTLS in the F_1_ hybrids (H1 × H8 and T485 × H8). **Table S10b** Names of the DEGFu-sp yield-related QTLS in the F_1_ hybrids (H1 × H8 and T485 × H8). **Table S10c** Names of the DEGFu-sp-related common QTLS in the F_1_ hybrids (H1 × H8 and T485 × H8). **Table S10d** Names of the DEGFu-sp-related common QTLS among the four F_1_ hybrids (H1 × H8, T485 × H8, T449 × H1 and T452 × H3). **Table S10e** Names and differential expressions of important DEGFu-sp genes in F_1_ hybrid (H1 × H8). **Table S10f** Names and differential expression of important DEGFu-sp genes in F_1_ hybrid (T485 × H8).

**Additional file 4: Table S11a, b** Gene IDs of DEGFu-sp-non-additive in the F_1_ hybrids. **Table S11c,d** Overlapped genes detected from DEGFu-sp-non-additive and cloned genes in F_1_ hybrids. **Table S12a** Overlapped genes detected from DEGFu-sp-cloned and mutant genes in H8 compared to H1. **Table S12b** Overlapped genes detected from DEGFu-sp-cloned and mutant genes in H8 compared to T485. **Table S12c** Overlapped genes detected from DEGFu-sp-cloned and all common mutant genes in H8 compared to H1 and T485. **Table S13a** Overlapped genes detected from DEGFu-sp and mutant genes in H8 compared to H1 and T485. **Table S13b** Overlapped genes detected from DEGFu-sp, mutant and cloned genes in H8 compared to H1 and T485. **Table S14** Predicted protein-protein interaction analysis of the cloned genes in DEGFu-sp. **Table S15** List of primers used for PCR and qRT-PCR*.* **Table S16** Name of 7 yield-related QTLs.

## Data Availability

All datasets supporting the conclusions of manuscript are provided in the main manuscript (Figures and Tables), additional files, and also deposited in publicly available repository (NCBI). The RNA-seq and Re-sequencing data are available from the NCBI under the accession number PRJNA576043 (https://www.ncbi.nlm.nih.gov/bioproject/PRJNA576043).

## Abbreviations

DEGFu-sp: Specific and differentially expressed genes uniquely belonging to F_1_ hybrid compared to parents
DEGs: Differentially expressed genes
HPH: High parent heterosis
MPH: Mid parent heterosis
TFs: Transcription factors
