# Supplementary material for: Heterosis analysis and underlying molecular regulatory mechanism in a wide-compatible neo-tetraploid rice line with long panicles
Source: BMC Plant Biol. 2020 Feb 21;20:83. doi: 10.1186/s12870-020-2291-z (PMC7035737; doi:10.1186/s12870-020-2291-z)
Supplement: Supplementary file 1 — Additional file 1: Figure S1a. Breeding procedure of Huaduo 8. Figure S1b Panicles of Huaduo 1 (H1), Huaduo 8 (H8) and their F1 hybrid. Figure S1c Plant appearance of parents and F1 hybrid generated by crossing with Huaduo 8 (H8). Figure S2 Original images of genotypes of parental lines at pollen sterility loci (Sa, Sb and Sc) and S5 locus detected by molecular markers. Figure S3 qRT-PCR validation for the quality of transcriptomic data. Figure S4 Hierarchical clustering analysis of all expressed genes based on transcriptome data. Figure S5 Prominent functional categories of genes in the eight tissues detected by Gene ontology enrichment analysis before and 5 days after flowering in two hybrids. Figure S6 Distribution of the common seven yield-related QTLs in the two F1 hybrids, T449 × H1 [17] and T452 × H3 [16], compared to the DEGFu-sp-related QTLs detected in the present study. Figure S7 Cluster analysis of the DNA variations in LP gene of Huaduo 8. Figure S8 Samples used for RNA-seq analysis. [file 12870_2020_2291_MOESM1_ESM.pdf]

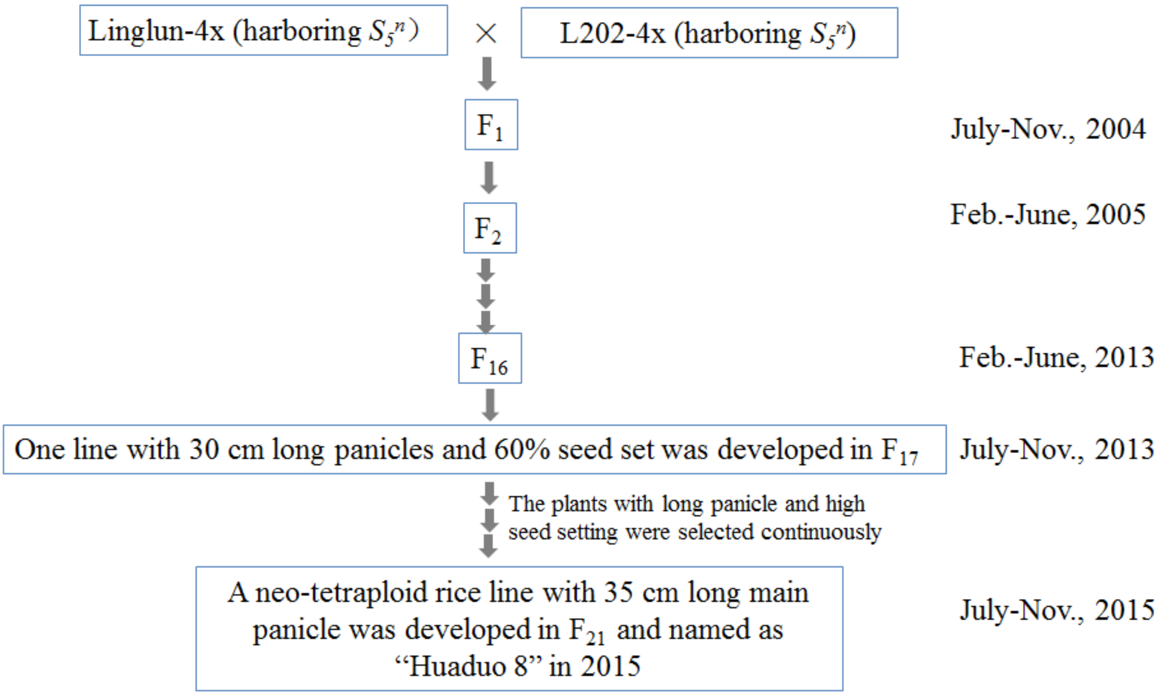

Fig. S1a. Breeding procedure of Huaduo 8

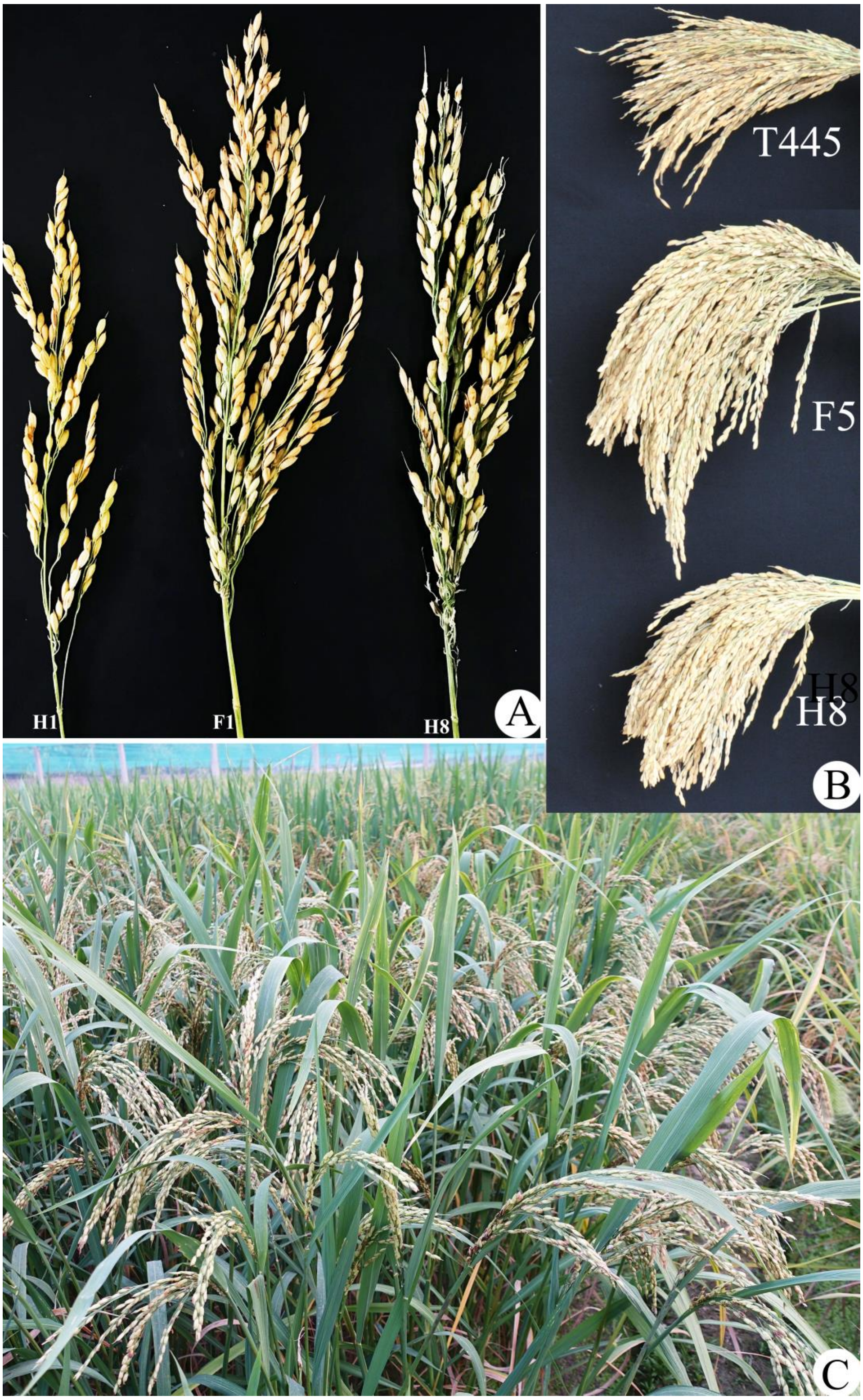

Fig. S1b Panicles of Huaduo 1 (H1), Huaduo 8 (H8) and their hybrid

A: Panicles of Huaduo 1 (H1), Huaduo 8 (H8) and their F_1 hybrid (F_1); B: Panicles of Luxiang 97-4x (T445, An autotetraploid rice), Huaduo 8 (H8) and their F_5 generation with high fertility; C: Panicles of Huaduo 3 × Huaduo 8 in F_3 generation

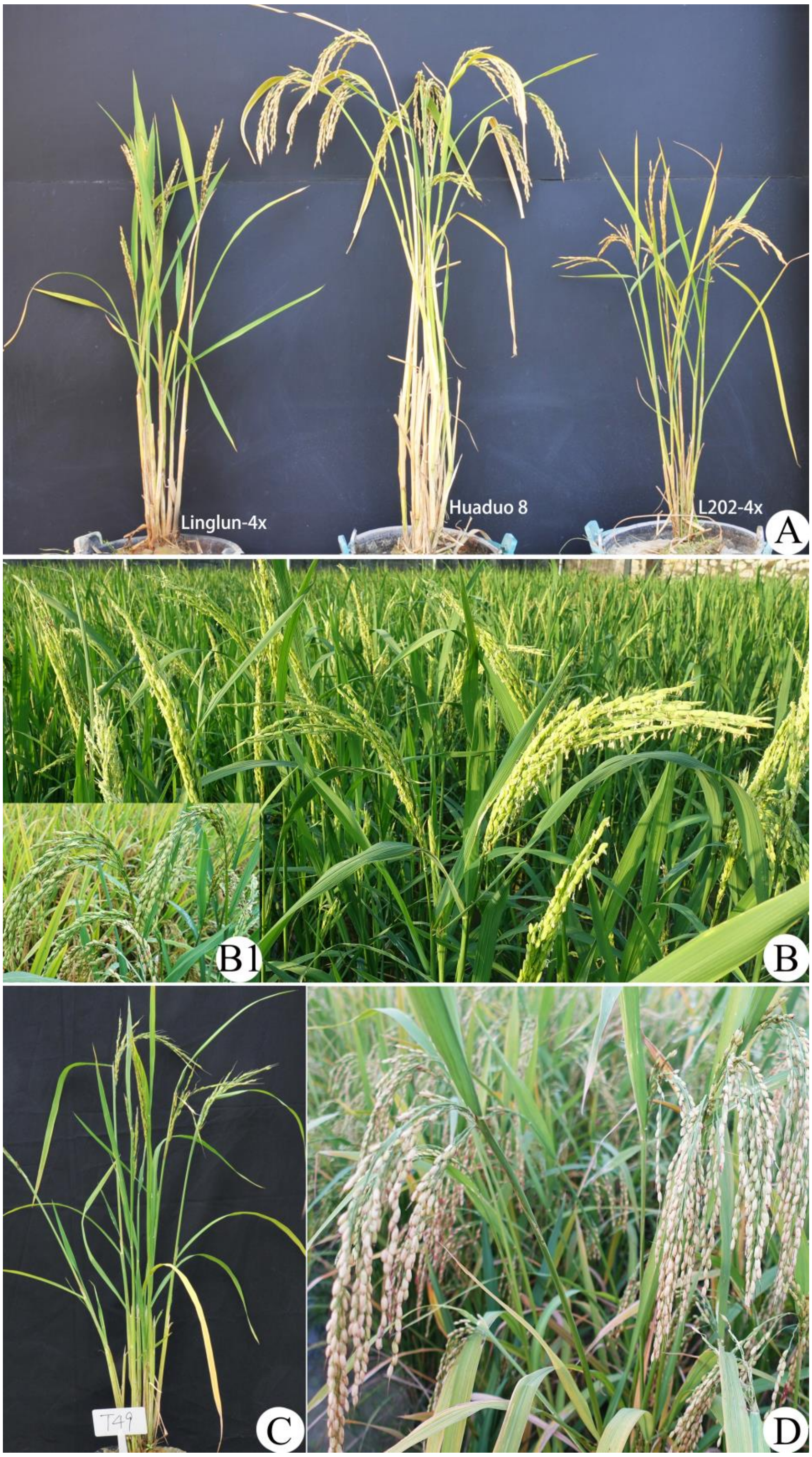

Fig. S1c Plant appearance of parents and F_1 hybrid generated by crossing with Huaduo 8 (H8)

[A: Linglun-4x (T428), Huaduo 8 (H8) and L202-4x (T43); B: Huaduo 8 at flowering stage, B1: Huaduo 8 at 15 days after flowering; C: Bengal-4x (T49, an autotetraploid rice with low fertility); D: F_1 of Bengal-4x × Huaduo 8]

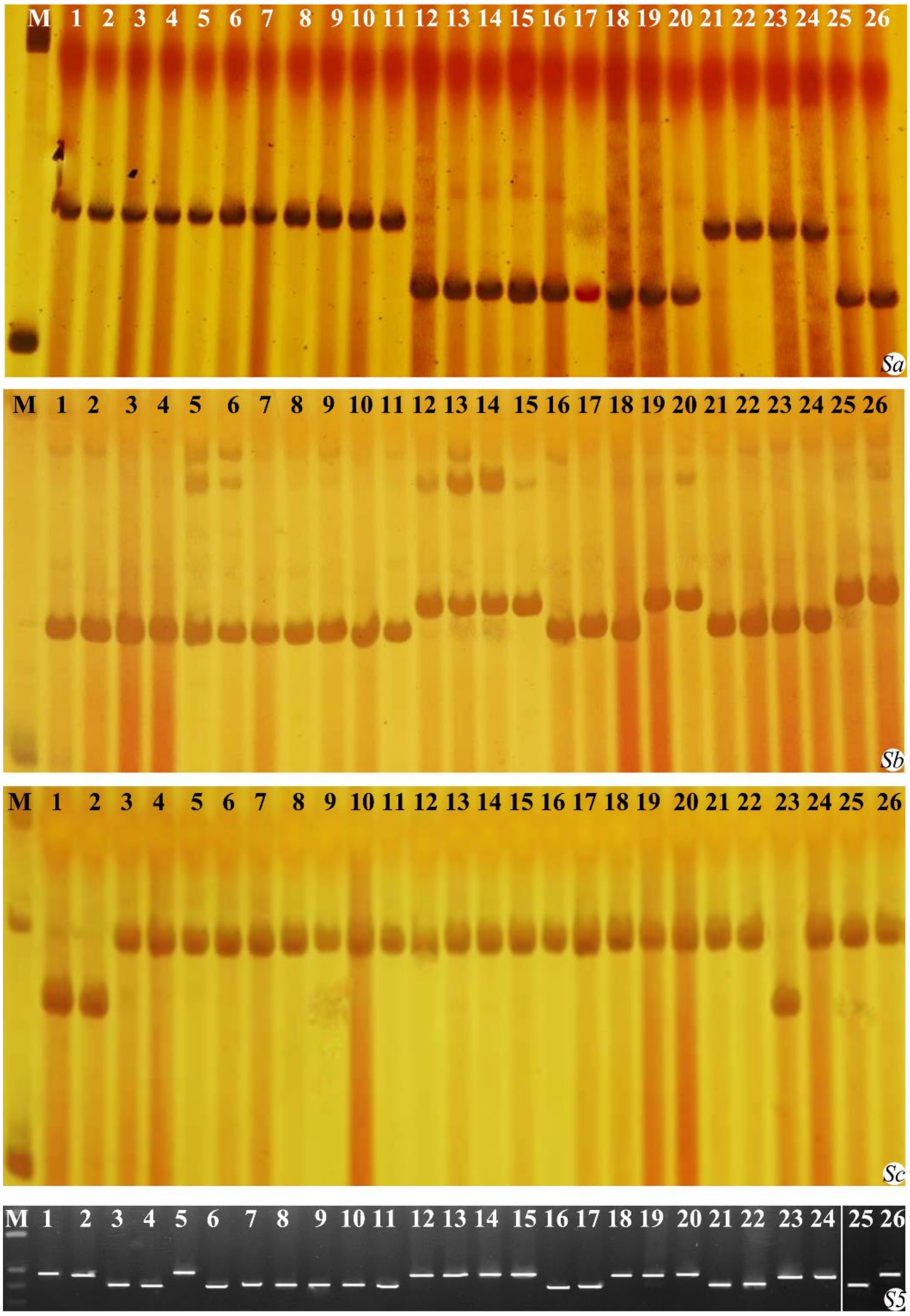

Fig. S2 Original images of genotypes of parental lines at pollen sterility loci (*Sa*, *Sb* and *Sc*) and *S5* locus detected by molecular markers.

First image indicates the genotypes at *Sa* locus, followed by (upper to lower) *Sb*, *Sc* and *S5* locus. M indicates DNA ladder, lanes 1-4 indicate control cultivars (typical *indica* and *japonica* cultivars), while lanes 5-26 indicate neo-tetraploid/autotetraploid rice lines (see the name of materials in Additional file 2: Table S1). Lanes 25 and 26 in S5 were taken from another gel.

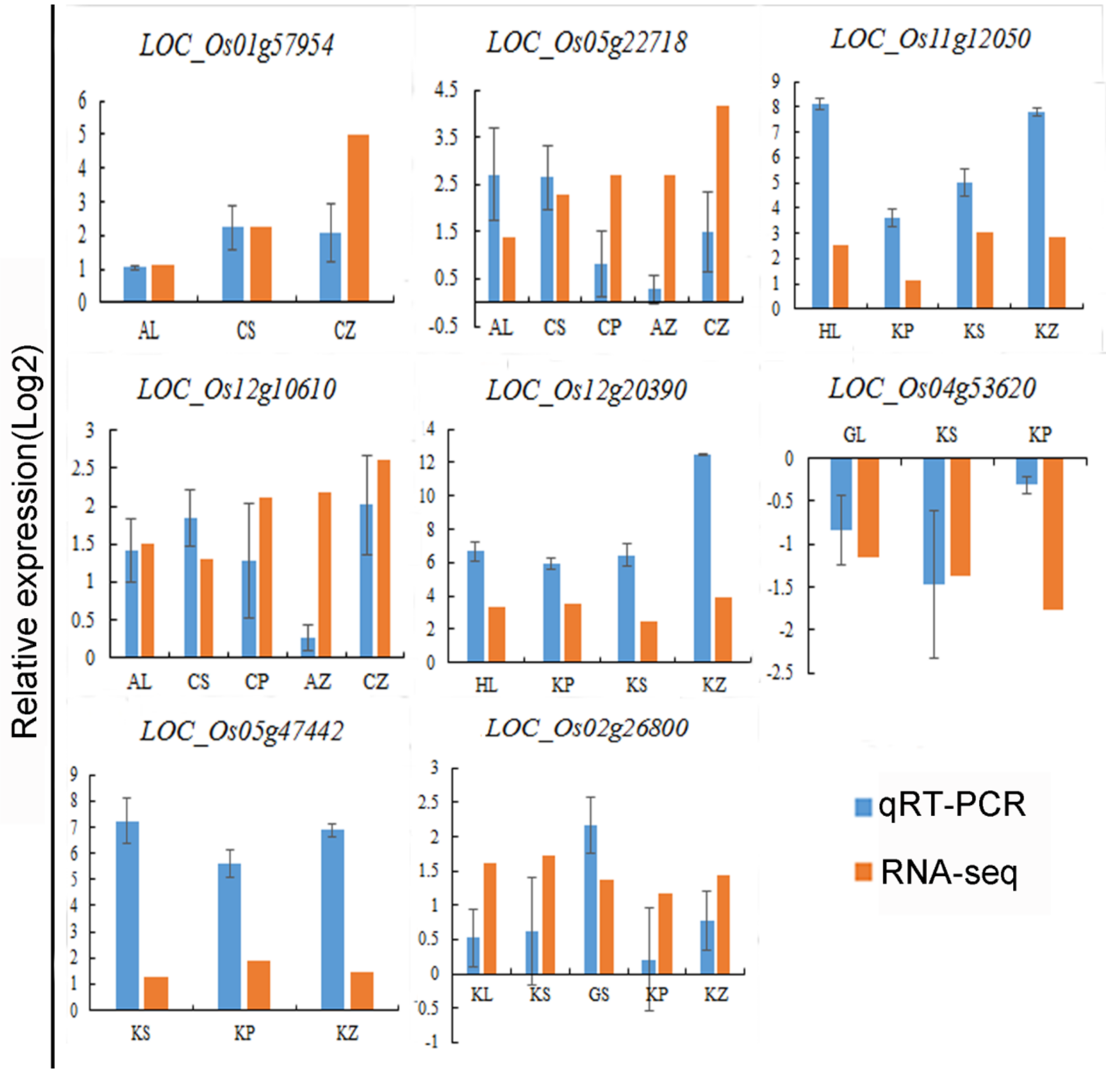

Fig. S3 qRT-PCR validation of the important genes detected in the present study (Codes of all X-axis are listed in Additional file 2: Table S4a)

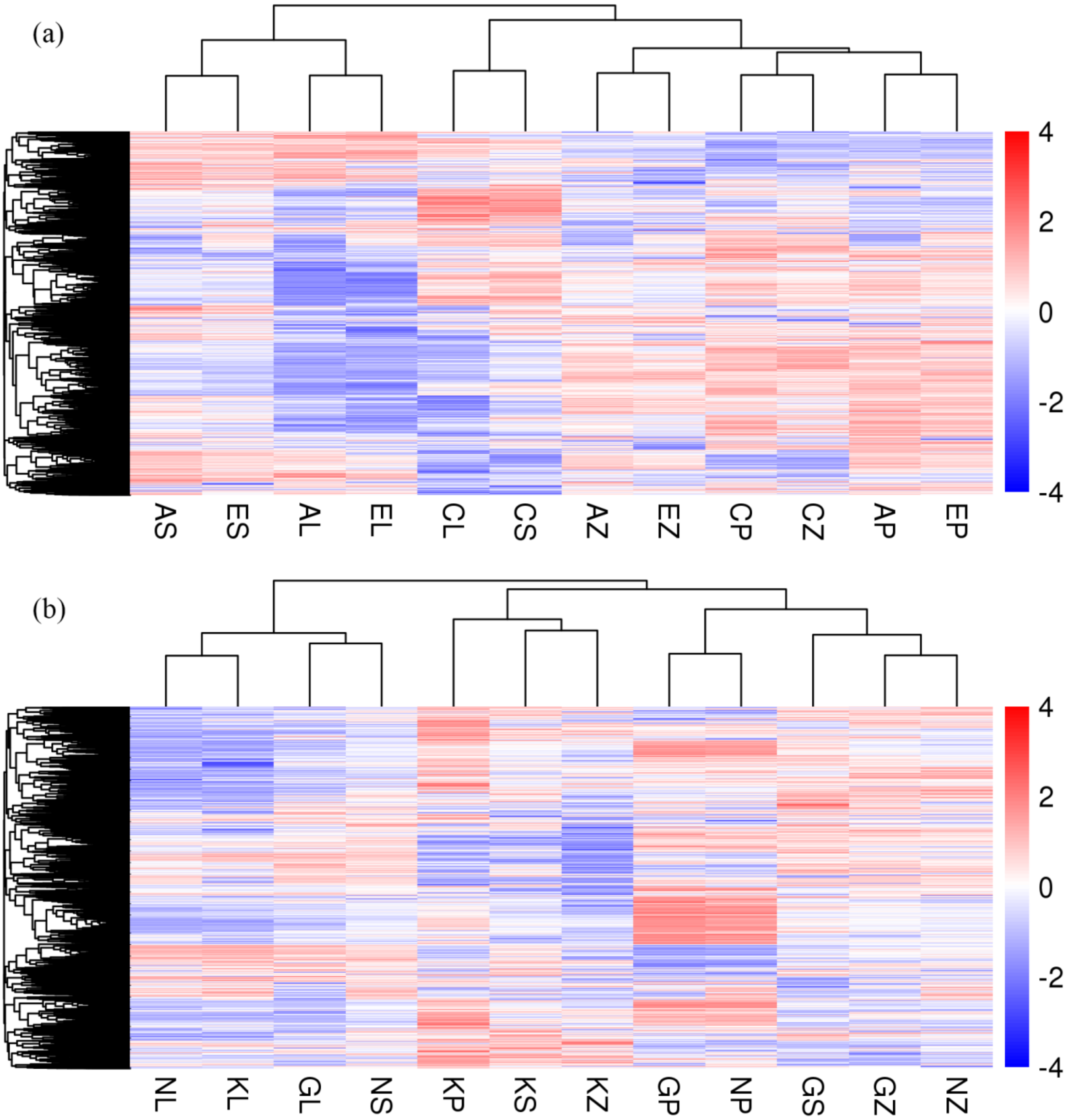

(a)
4
2
0
-2
-4
AS
ES
AL
EL
CL
CS
AZ
EZ
CP
CZ
AP
EP
(b)
4
2
0
-2
-4
NL
KL
GL
NS
KP
KS
KZ
GP
NP
GS
GZ
NZ

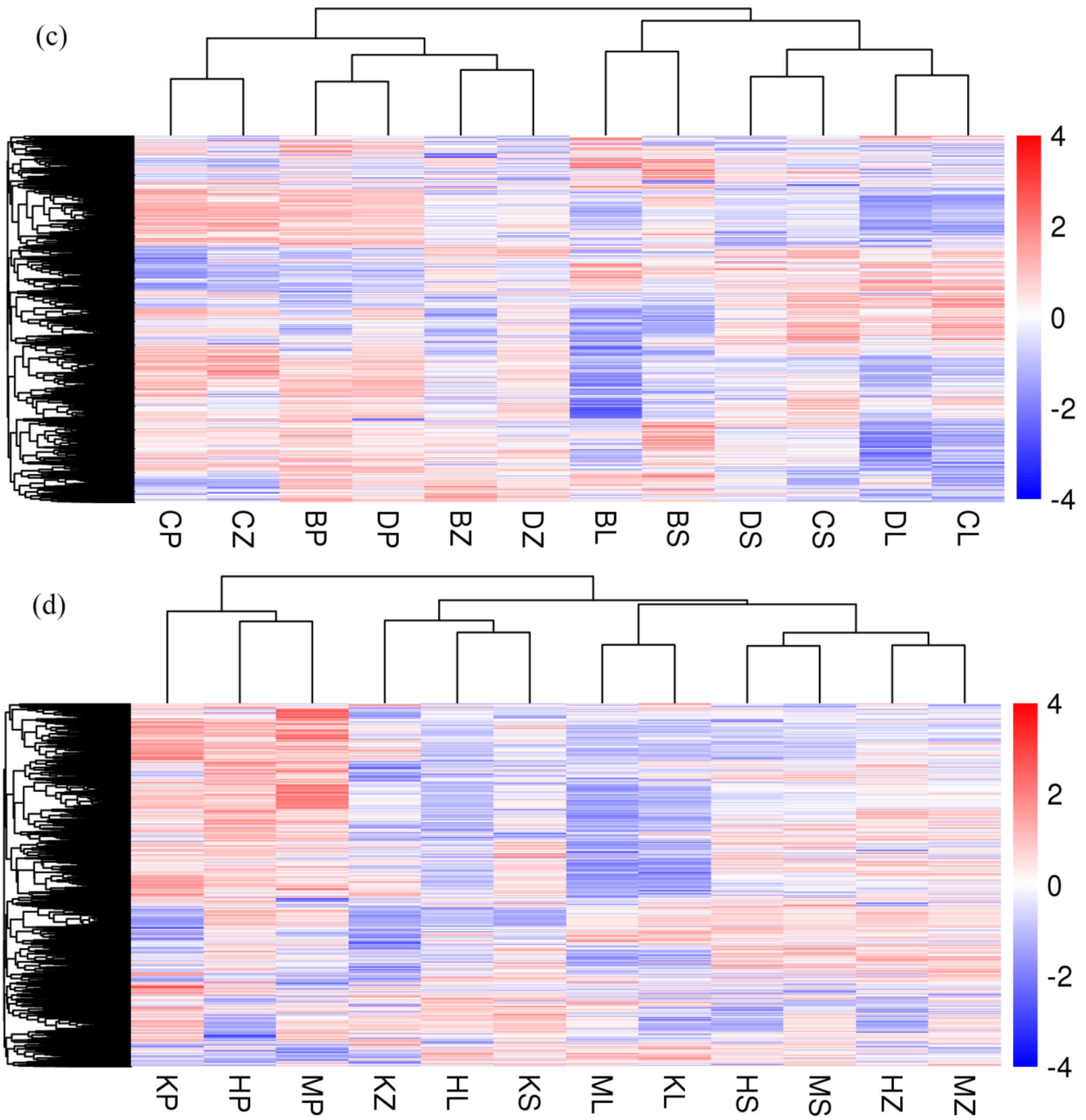

Fig. S4. Hierarchical clustering analysis of all expressed genes in F_1 hybrid and their parents based on transcriptome data

(a): F_1 hybrid (H1×H8) and its parents before flowering; (b): F_1 hybrid (H1×H8) and its parents 5 days after flowering; (c): F_1 hybrid (T485×H8) and its parents before flowering; (d) F_1 hybrid (T485×H8) and its parents 5 days after flowering. Codes of all X-axis are listed in Additional file 2: Table S4a

Hybrids		(H1xH8)F_1								(T485xH8)F_1							
**A. Biological process**		L_0	S_0	P_0	Z_0	L_5	S_5	P_5	Z_5	L_0	S_0	P_0	Z_0	L_5	S_5	P_5	Z_5
GO:0055114	oxidation reduction		■	■	■		■	■	■	■	■	■			■		
GO:0005975	carbohydrate metabolic process		■	■	■		■			■		■		■		■	
GO:0044267	cellular protein metabolic process	■	■				■		■	■			■			■	■
GO:0016310	phosphorylation			■	■		■		■	■					■		■
GO:0006796	phosphate metabolic process			■	■		■		■	■					■		■
GO:0006793	phosphorus metabolic process			■	■		■		■	■					■		■
GO:0065007	biological regulation	■	■						■	■				■	■		
GO:0043687	post-translational protein modification			■			■		■	■					■		■
GO:0006464	protein modification process			■			■		■	■					■		■
GO:0043412	macromolecule modification			■			■		■	■					■		■
GO:0006468	protein amino acid phosphorylation			■			■		■	■					■		■
GO:0050794	regulation of cellular process	■	■							■				■	■		
GO:0010467	gene expression	■	■										■	■		■	
GO:0050789	regulation of biological process	■	■							■				■	■		
GO:0006350	transcription		■											■	■	■	
GO:0044262	cellular carbohydrate metabolic process									■				■		■	
GO:0006810	transport						■			■							■
GO:0051234	establishment of localization						■			■							■
GO:0051179	localization						■			■							■
GO:0043436	oxoacid metabolic process												■		■	■	
GO:0019752	carboxylic acid metabolic process												■		■	■	
GO:0006082	organic acid metabolic process												■		■	■	
GO:0042180	cellular ketone metabolic process												■		■	■	
GO:0009308	amine metabolic process												■		■	■	
GO:0044281	small molecule metabolic process		■										■			■	
GO:0015979	photosynthesis					■					■	■					
GO:0065004	protein-DNA complex assembly	■												■		■	
GO:0006323	DNA packaging	■												■		■	
GO:0034728	nucleosome organization	■												■		■	
GO:0065003	macromolecular complex assembly	■												■		■	
GO:0043933	macromolecular complex subunit organization	■												■		■	
GO:0019538	protein metabolic process								■				■			■	
GO:0022607	cellular component assembly	■												■		■	
GO:0006334	nucleosome assembly	■												■		■	
GO:0034621	cellular macromolecular complex subunit organization	■												■		■	
GO:0034622	cellular macromolecular complex assembly	■												■		■	
GO:0031497	chromatin assembly	■												■		■	
GO:0071103	DNA conformation change	■												■		■	
GO:0006412	translation	■											■			■	
GO:0031326	regulation of cellular biosynthetic process													■	■		
GO:0080090	regulation of primary metabolic process													■	■		
GO:0009889	regulation of biosynthetic process													■	■		
GO:0010468	regulation of gene expression													■	■		
GO:0045449	regulation of transcription													■	■		
GO:0051171	regulation of nitrogen compound metabolic process													■	■		
GO:0060255	regulation of macromolecule metabolic process													■	■		
GO:0019219	regulation of nucleobase, nucleoside, nucleotide and nucleic acid metabolic process													■	■		
GO:0031323	regulation of cellular metabolic process													■	■		
GO:0010556	regulation of macromolecule biosynthetic process													■	■		
GO:0019222	regulation of metabolic process													■	■		
GO:0055085	transmembrane transport						■			■							
GO:0006520	cellular amino acid metabolic process												■			■	
GO:0034641	cellular nitrogen compound metabolic process												■			■	
GO:0005976	polysaccharide metabolic process		■	■													
GO:0006519	cellular amino acid and derivative metabolic process												■			■	
GO:0006811	ion transport				■					■							
GO:0044106	cellular amine metabolic process												■			■	
GO:0006812	cation transport				■					■							
GO:0009607	response to biotic stimulus			■		■											
GO:0016051	carbohydrate biosynthetic process		■											■			
GO:0034637	cellular carbohydrate biosynthetic process		■											■			
GO:0046394	carboxylic acid biosynthetic process												■			■	
GO:0044283	small molecule biosynthetic process												■			■	
GO:0044085	cellular component biogenesis													■		■	
GO:0016053	organic acid biosynthetic process												■			■	
GO:0034660	ncRNA metabolic process												■			■	
GO:0006457	protein folding												■			■	
**B. Molecular function**		L_0	S_0	P_0	Z_0	L_5	S_5	P_5	Z_5	L_0	S_0	P_0	Z_0	L_5	S_5	P_5	Z_5
GO:0016491	oxidoreductase activity	■	■	■	■		■	■	■	■	■	■			■		■
GO:0016773	phosphotransferase activity, alcohol group as acceptor			■	■		■		■	■					■		■
GO:0004672	protein kinase activity			■	■		■		■	■					■		■
GO:0004674	protein serine/threonine kinase activity			■	■		■		■	■					■		■
GO:0016301	kinase activity						■		■	■					■		■
GO:0030554	adenyl nucleotide binding			■			■		■	■							■
GO:0001883	purine nucleoside binding			■			■		■	■							■
GO:0001882	nucleoside binding			■			■		■	■							■
GO:0000166	nucleotide binding						■		■	■						■	■
GO:0016798	hydrolase activity, acting on glycosyl bonds			■	■			■				■				■	
GO:0004553	hydrolase activity, hydrolyzing O-glycosyl compounds			■	■			■				■				■	
GO:0032559	adenyl ribonucleotide binding						■		■	■							■
GO:0005524	ATP binding						■		■	■							■
GO:0003700	transcription factor activity	■							■					■	■		
GO:0032553	ribonucleotide binding						■		■	■							■
GO:0017076	purine nucleotide binding						■		■	■							■
GO:0032555	purine ribonucleotide binding						■		■	■							■
GO:0009055	electron carrier activity			■	■			■		■							
GO:0003735	structural constituent of ribosome	■	■										■			■	
GO:0005198	structural molecule activity	■	■										■			■	
GO:0030528	transcription regulator activity	■	■												■		
GO:0005215	transporter activity			■			■			■							
GO:0020037	heme binding			■				■							■		
GO:0005506	iron ion binding			■				■							■		
GO:0046906	tetrapyrrole binding			■				■							■		
GO:0043565	sequence-specific DNA binding	■					■			■							
GO:0048037	cofactor binding	■		■	■												
GO:0004497	monooxygenase activity			■				■							■		
GO:0016757	transferase activity, transferring glycosyl groups						■								■		
GO:0045735	nutrient reservoir activity								■							■	
GO:0050662	coenzyme binding	■			■												
**C. Cell component**		L_0	S_0	P_0	Z_0	L_5	S_5	P_5	Z_5	L_0	S_0	P_0	Z_0	L_5	S_5	P_5	Z_5
GO:0016020	membrane			■	■	■	■			■	■	■	■	■			■
GO:0032991	macromolecular complex	■	■			■				■	■	■	■	■		■	
GO:0005623	cell										■	■	■	■		■	
GO:0044464	cell part										■	■	■	■		■	
GO:0044425	membrane part						■			■		■		■			
GO:0005840	ribosome	■	■										■			■	
GO:0030529	ribonucleoprotein complex	■	■										■			■	
GO:0032993	protein-DNA complex	■				■								■		■	
GO:0000786	nucleosome	■				■								■		■	
GO:0034357	photosynthetic membrane					■					■	■					
GO:0009579	thylakoid					■					■	■					
GO:0043234	protein complex					■				■	■						
GO:0005737	cytoplasm	■											■			■	
GO:0044444	cytoplasmic part	■											■			■	
GO:0009521	photosystem					■					■	■					
GO:0016021	integral to membrane						■					■					
GO:0031224	intrinsic to membrane						■					■					
GO:0044424	intracellular part												■			■	
GO:0015935	small ribosomal subunit												■			■	
GO:0005622	intracellular												■			■	
GO:0043229	intracellular organelle												■			■	
GO:0043232	intracellular non-membrane-bounded organelle												■			■	
GO:0009522	photosystem I					■					■						
GO:0015934	large ribosomal subunit												■			■	
GO:0043228	non-membrane-bounded organelle												■			■	
GO:0033279	ribosomal subunit												■			■	
GO:0043226	organelle												■			■	

Fig. S5. Prominent functional categories of genes in the eight tissues detected by Gene ontology enrichment analysis before and 5 days after flowering in two hybrids

L_0, Flag leaf; S_0, Leaf sheath; P_0, Spikelet; Z_0, Main panicle axis before flowering; L_5, Flag leaf; S_5, Leaf sheath; P_5, Spikelet; Z_5, Main panicle axis 5 days after flowering

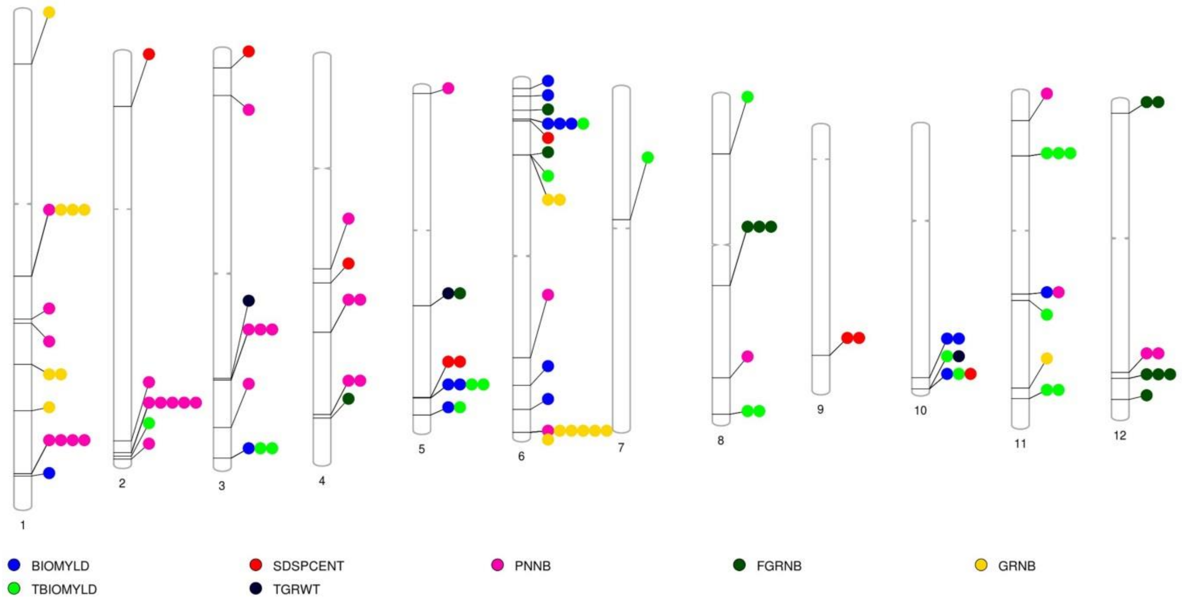

Fig. S6 Distribution of the common seven yield-related QTLs in our two F_1 hybrids, T449 × H1 [17] and T452 × H3 [16], compared to the DEGFu-sp-related QTLs detected in the present study.

Seven rice yield-related QTLs included biomass yield (TBIOMYLD), biomass yield (BIOMYLD), filled grain number (FGRNB), grain number (GRNB), panicle number (PNNB), seed set percent (SDSPCENT) and 1000-grain weight (TGRWT).

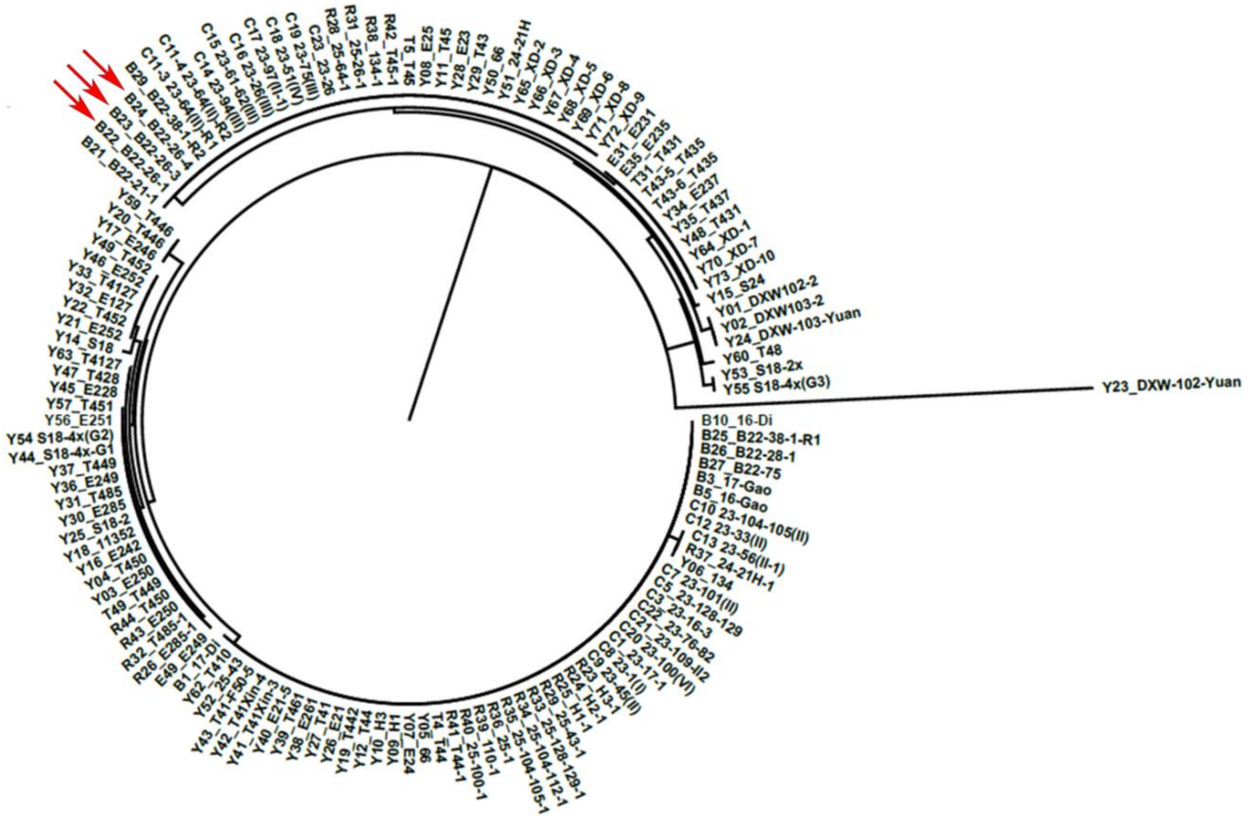

Fig. S7. Cluster of the DNA variations in LP gene of Huaduo 8

Arrows indicated Huaduo 8, in which DNA sequence was different from most of the other neo-tetraploid, autotetraploid and diploid rice lines. Three different plants of Huaduo 8 were re-sequenced

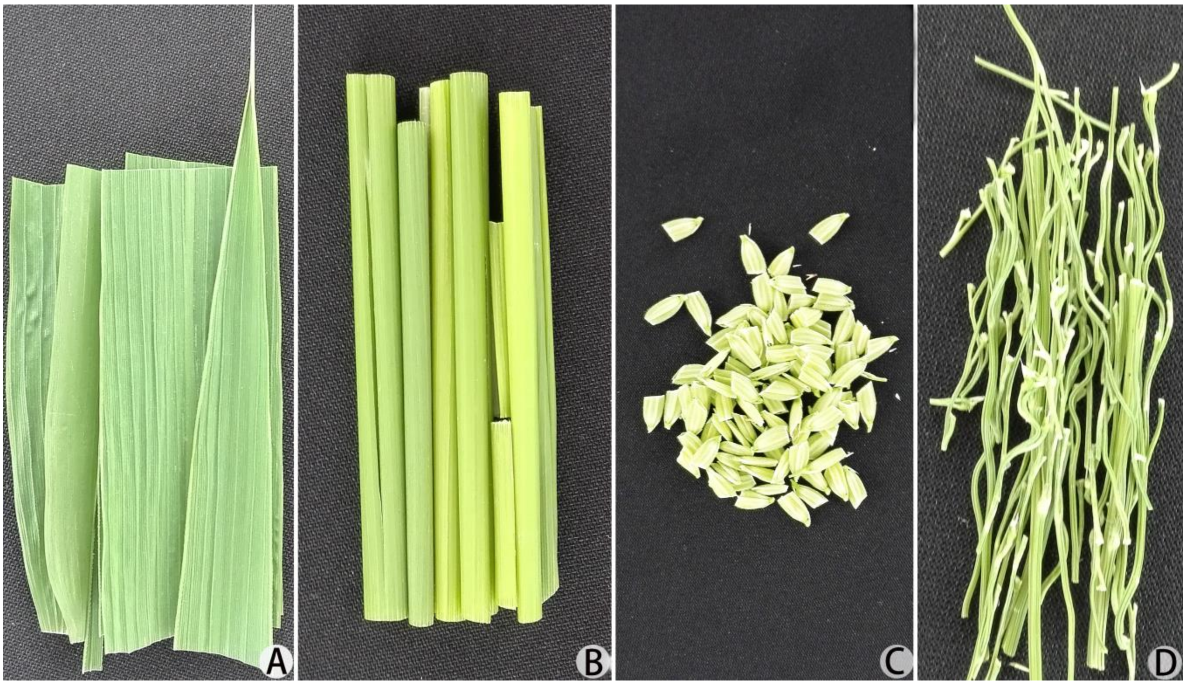

Fig. S8 Samples used for RNA-seq analysis

A. Flag leaf; B. Leaf sheath; C. Spikelets; D. Main panicle axis
